# A SlERF4–SlTPP1 module enhances drought tolerance in tomato by increasing the root/shoot ratio

**DOI:** 10.1093/hr/uhag070

**Published:** 2026-03-02

**Authors:** Heng Wang, Lin Chai, Hongjun Yu, Hongxue Li, Debao Yi, Sufian Ikram, Tao Lu, Yang Li, Xueyong Yang, Weijie Jiang, Qiang Li

**Affiliations:** State Key Laboratory of Vegetable Biobreeding, Institute of Vegetables and Flowers, Chinese Academy of Agricultural Sciences, Beijing 100081, China; State Key Laboratory of Vegetable Biobreeding, Institute of Vegetables and Flowers, Chinese Academy of Agricultural Sciences, Beijing 100081, China; State Key Laboratory of Vegetable Biobreeding, Institute of Vegetables and Flowers, Chinese Academy of Agricultural Sciences, Beijing 100081, China; College of Plant Science and Technology, Beijing University of Agriculture, Beijing 102206, China; State Key Laboratory of Vegetable Biobreeding, Institute of Vegetables and Flowers, Chinese Academy of Agricultural Sciences, Beijing 100081, China; State Key Laboratory of Vegetable Biobreeding, Institute of Vegetables and Flowers, Chinese Academy of Agricultural Sciences, Beijing 100081, China; State Key Laboratory of Vegetable Biobreeding, Institute of Vegetables and Flowers, Chinese Academy of Agricultural Sciences, Beijing 100081, China; State Key Laboratory of Vegetable Biobreeding, Institute of Vegetables and Flowers, Chinese Academy of Agricultural Sciences, Beijing 100081, China; State Key Laboratory of Vegetable Biobreeding, Institute of Vegetables and Flowers, Chinese Academy of Agricultural Sciences, Beijing 100081, China; State Key Laboratory of Vegetable Biobreeding, Institute of Vegetables and Flowers, Chinese Academy of Agricultural Sciences, Beijing 100081, China; College of Horticulture, Xinjiang Agricultural University, Urumqi 830052, China; State Key Laboratory of Vegetable Biobreeding, Institute of Vegetables and Flowers, Chinese Academy of Agricultural Sciences, Beijing 100081, China

## Abstract

Drought tolerance is a pivotal trait for tomato (*Solanum lycopersicum*) genetic improvement, and enhancing the root/shoot ratio (R/S) serves as a core adaptive strategy for plants to cope with water deficit. While trehalose-6-phosphate phosphatase (TPP) genes are implicated in plant drought responses, their role in modulating R/S remains unclear. Here, we characterized *SlTPP1* as a key positive regulator of drought tolerance in tomato. We found that drought stress dynamically induces *SlTPP1* expression in roots while suppressing it in leaves. Mechanistically, *SlTPP1* overexpression increases root soluble sugar content and upregulates night-specific expression of cell wall biosynthesis genes in roots to promote root growth, while concurrently suppressing the ethylene signaling pathway in leaves to increase R/S. Furthermore, we identified the transcription factor *SlERF4* as a direct upstream repressor of *SlTPP1*. SlERF4 binds to the CE1 element (CACCG) in the *SlTPP1* promoter and inhibits its transcription. CRISPR/Cas9-mediated knockout of *SlERF4* results in enhanced drought tolerance, elevated *SlTPP1* expression, increased R/S, and upregulation of root cell wall biosynthesis genes. Additionally, drought enhances ethylene biosynthesis in tomato leaves while concurrently reducing that in roots. Collectively, our study unveils a novel SlERF4–SlTPP1 regulatory module that enhances drought tolerance in tomato through the regulation of R/S, providing strategic targets for breeding drought-tolerant crops.

## Introduction

Drought stress is one of the most devastating abiotic stresses, severely limiting global crop productivity and food security [1, 2]. With climate change exacerbating the frequency and intensity of drought events, enhancing drought tolerance in crops has become a critical goal for sustainable horticulture [3, 4]. Recent advances have uncovered multifaceted strategies, ranging from stomatal optimization and reactive oxygen species (ROS) scavenging to hormonal crosstalk and metabolic reprogramming [5–9]. In addition, promoting root growth and increasing the root/shoot ratio (R/S) are key strategies to optimize water acquisition while minimizing transpirational loss.

Global field studies have consistently shown that drought enhances R/S by altering root morphology, with reductions in root length but increases in root diameter and cortical aerenchyma, leading to a relative maintenance of root biomass compared to shoot biomass [10]. This phenomenon is widely observed across diverse species, including potato, where drought elevates R/S by preferentially maintaining root growth over shoot growth [11]; tomato, under salinity-induced drought analog, where hormonal shifts promote root allocation [12]; rice, through altered carbohydrate partitioning and enhanced enzyme activities like sucrose-phosphate synthase in leaves and invertase in roots [13]; *Arabidopsis*, with increased carbon export to roots via sucrose transporters [14]; wheat, where wild emmer introgression lines modify R/S dynamics under water stress [15, 16]; and sorghum, via transcriptional regulation of lateral root development [17]. Physiologically, drought induces opposing metabolic responses in shoots and roots: shoots downregulate growth-related metabolism (e.g. reduced sugars, amino acids, and nutrient concentrations), while roots upregulate metabolic activity to support water and nutrient uptake, thereby reallocating resources to belowground organs [18]. Hormonal coordination is central to this process; abscisic acid (ABA) accumulates rapidly in roots and leaves under stress, suppressing shoot growth by reducing cytokinins and altering auxin distribution, while ethylene modulation—exemplified in sorghum by SbWRKY50 transcription factor repressing ACS genes to inhibit ethylene synthesis—promotes root expansion [12, 17]. At the molecular level, drought and ABA activate SnRK2 kinases that phosphorylate sucrose transporters (e.g. SWEET11 and SWEET12 in *Arabidopsis*), enhancing their oligomerization and activity to facilitate sucrose flux from shoots to roots, thereby increasing root sugar accumulation and R/S [19]. This is complemented by upregulation of sucrose transporter genes (e.g. AtSUC2 and AtSWEETs) in both shoots and roots, ensuring efficient phloem loading and unloading [14]. Genetic approaches further validate R/S manipulation for drought resilience; for instance, wild emmer wheat introgressions in durum wheat alter root-to-shoot carbon reallocation, improving water influx and gas exchange under low vapor pressure deficit conditions [15, 16], while selection for high R/S in tall fescue populations enhances field drought tolerance, confirming its utility in breeding programs [20]. Understanding these integrated mechanisms—spanning phytohormone signaling, carbon partitioning, and root system architecture remodeling—provides a foundation for developing drought-tolerant crops through targeted R/S regulation.

The trehalose-6-phosphate phosphatase (TPP) gene family, which catalyzes the dephosphorylation of trehalose-6-phosphate (T6P) to produce trehalose, plays a pivotal role in plant stress responses by integrating sugar signaling with growth and development [21, 22]. Recent studies have demonstrated that TPP genes function as positive regulators of drought tolerance across various plant species, including *Arabidopsis*, maize, and tobacco. The mechanisms underlying TPP-mediated drought resilience involve multiple pathways: (i) modulation of sugar metabolism, where TPP overexpression leads to accumulation of trehalose, sucrose, and total soluble sugars, acting as osmolytes to maintain cellular homeostasis under water deficit [23–25]; (ii) regulation of stomatal movement via ABA signaling, resulting in reduced stomatal aperture and improved water use efficiency (WUE) [26, 27]; (iii) enhancement of root architecture, such as increased primary root length, which facilitates water uptake from deeper soil layers [26–28]; (iv) transcriptional activation by stress-responsive factors like DREB1A and ABF/AREBs, which directly bind to TPP promoters to induce expression under drought conditions [24, 27]; and (v) mitigation of oxidative damage by lowering ROS levels through soluble sugar-mediated scavenging [24, 25]. For instance, overexpression of AtTPPF in *Arabidopsis* boosted drought tolerance by elevating soluble sugar content and reducing H₂O₂ accumulation [24], while *OsTPP1* expression in maize ears improved yield under drought by optimizing carbon partitioning and sucrose levels [23]. Similarly, GhTPPA2 in tobacco enhanced drought resilience by promoting sugar accumulation and carbon fixation [25]. These findings collectively underscore the potential of TPP genes as key targets for breeding drought-tolerant crops. However, the specific mechanisms by which TPP influences root growth and R/S remain to be elucidated.

The AP2/ERF (APETALA2/ethylene-responsive factor) family of transcription factors plays a pivotal role in enhancing plant drought tolerance by orchestrating diverse molecular and physiological responses [29–33]. Extensive studies have illustrated that ERFs modulate plant adaptation to drought stress via multiple regulatory mechanisms, including wax biosynthesis [31, 34], ROS scavenging [35], osmotic adjustment [36, 37], and the mediation of hormone signaling pathways [30, 33]. For instance, NtERF172 in tobacco directly activates catalase (NtCAT) expression to mitigate H₂O₂ accumulation under drought stress [35], while TaERF87 in wheat synergistically promotes proline biosynthesis via TaP5CS1/TaP5CR1 to maintain cellular homeostasis [37]. Similarly, ERFs like MdERF38 and *Arabidopsis* DREB26/ERF12 enhance drought tolerance by modulating anthocyanin synthesis and cuticular wax deposition, respectively, thereby reducing water loss and oxidative damage [31, 38]. In contrast to these well-characterized aerial responses, the role of ERFs in modulating root architecture and R/S, a critical trait for water acquisition under drought conditions, remains underexplored.

Tomato (*Solanum lycopersicum*) is not only a globally important vegetable crop but also a well-established model system for studying fruit biology and stress physiology [39]. Although the tomato genome encodes a family of TPP genes [21], the functional characterization of individual members, especially their role in regulating root system architecture and drought tolerance through cross-talk with hormonal pathways, is far from complete. This study elucidates the function and molecular mechanism of *SlTPP1* in positively regulating drought tolerance in tomato, which enhances root growth by increasing root sugar content and upregulating cell wall synthesis genes, while elevating the R/S via repressing ethylene signal transduction pathway genes in leaves. In addition, the transcription factor SlERF4 directly binds to the *SlTPP1* promoter to negatively regulate its expression, and *SlERF4* exerts a negative effect on tomato drought tolerance by modulating the R/S. Collectively, our findings uncover a SlERF4–SlTPP1 module that improves tomato drought tolerance through the modulation of the R/S.

## Results

### The *SlTPP1* gene responds to drought stress in tomato

**Figure 1:**
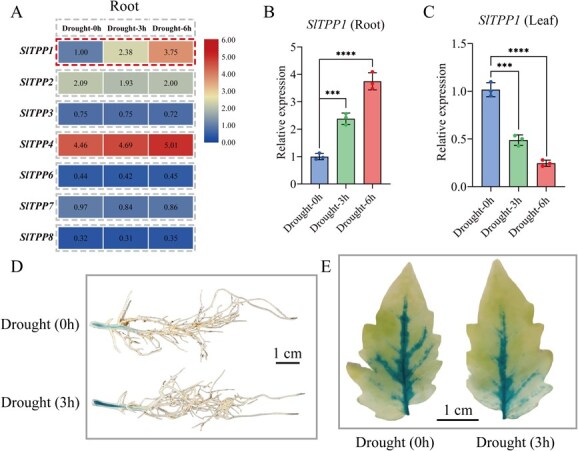
*SlTPP1* expression in tomato roots (induction) and leaves (repression) under drought stress. (A) Expression profiles of *SlTPP* genes in tomato roots under drought stress at 0, 3, and 6 h. The values in the boxes indicate the relative expression level of each gene. (B, C) qRT-PCR analysis of *SlTPP1* expression dynamics in tomato roots (B) and leaves (C) at 3 and 6 h post-drought treatment. Data are shown as means ± SD (*n* = 3). Statistical significance was analyzed using two-tailed Student’s *t* test (^*^P < 0.05, ^**^P < 0.01, ^***^P < 0.001, ^****^P < 0.0001). (D, E) GUS staining of roots (D) and leaves (E) in proSlTPP1-GUS transgenic tomato plants at 3 h post-drought treatment. Bars, 1 cm.

Eight *SlTPP* genes were identified in tomato (*S. lycopersicum*) [22, 40]. To explore the roles of *SlTPP* family members in regulating tomato drought tolerance, we first analyzed the expression dynamics of *SlTPP* genes in roots and leaves under drought stress via quantitative real-time PCR (qRT-PCR). Our results demonstrated that in tomato roots, *SlTPP1* expression was significantly induced by drought stress, whereas the expression of the remaining *SlTPP* genes exhibited no significant alterations at 3 and 6 h post-drought stress (Fig. 1A and B). In contrast, drought stress resulted in a marked downregulation of *SlTPP1* expression in leaves, while *SlTPP3* expression was modestly upregulated (Fig. S1; Fig. 1C). Furthermore, we exposed proSlTPP1-GUS transgenic tomato seedlings to drought stress prior to GUS staining. In line with the qRT-PCR data, drought stress induced *SlTPP1* expression in roots while repressing its expression in leaves (Fig. 1D and E). Collectively, these findings indicate that *SlTPP1* may play a pivotal role in mediating tomato responses to drought stress.

### *SlTPP1* positively regulates drought tolerance by increasing R/S in tomato

**Figure 2:**
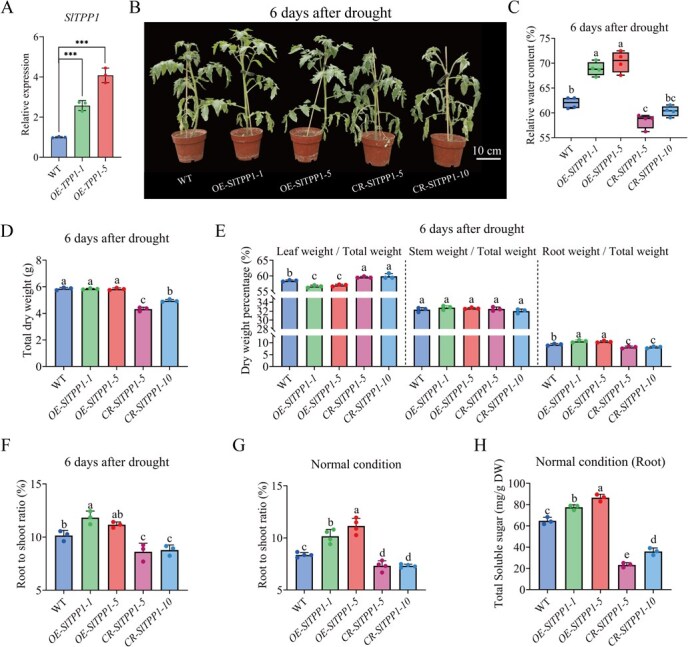
*SlTPP1* positively regulates drought tolerance by increasing R/S in tomato. (A) qRT-PCR analysis of the *SlTPP1* gene expression level in *OE-SlTPP1* tomato plants. Data are means ± SD (*n* = 3). Statistical significance was analyzed using two-tailed Student’s *t* test (^*^P < 0.05, ^**^P < 0.01, ^***^P < 0.001, ^****^P < 0.0001). (B) Phenotypes of WT, *OE-SlTPP1* and *CR-SlTPP1* tomato seedlings after 6 days of drought treatment. Bar, 10 cm. (C–F) Analysis of relative leaf water content (C), total DW (D), DW percentage (E), and R/S (F) in WT, *OE-SlTPP1*, and *CR-SlTPP1* tomato seedlings after 6 days of drought treatment. (G, H) Analysis of R/S (G) and total soluble sugar content in roots (H) in WT, *OE-SlTPP1*, and *CR-SlTPP1* tomato seedlings under normal conditions. (C–H) Data are means ± SD (*n* = 3 to 4), and significance was analyzed by one-way ANOVA followed by Tukey’s test. Different lowercase letters indicate a significant difference (*P* < 0.05).

To investigate the function of *SlTPP1* in drought tolerance of tomato, we generated *SlTPP1*-overexpressing tomato lines, *OE-TPP1-1* and *OE-TPP1-5*. qRT-PCR results showed that the expression level of *SlTPP1* in *OE-TPP1* tomato plants was significantly higher than that in WT (Fig. 2A). Meanwhile, we also obtained two *SlTPP1* gene-edited lines, and the specific editing strategy has been described in our previous study [20]. Drought stress was simulated by watering 5-week-old tomato seedlings of each genotype with 15% (w/v) PEG6000. Phenotypic analysis after 6 days revealed that compared with WT, the wilting degree of *OE-TPP1* was significantly alleviated, while that of *CR-TPP1* was more severe (Fig. 2B). Consistent with the wilting degree, the relative water content (RWC) in leaves of *OE-TPP1* tomatoes was significantly higher than that in WT, whereas that in *SlTPP1* mutants was lower than that in WT (Fig. 2C). These results indicate that *SlTPP1* positively regulates drought tolerance in tomato.

To further explore the mechanism by which *SlTPP1* regulates drought tolerance, we first analyzed the total dry weight (DW) and dry matter allocation of plants of each genotype after drought treatment. The results showed that after 6 days of drought treatment, there was no significant difference in total DW between *OE-TPP1* and WT, while the total DW of *CR-TPP1* was significantly lower than that of WT (Fig. 2D). The results of dry matter allocation indicated that, compared with WT, overexpression of *SlTPP1* significantly increased dry matter allocation to roots and decreased allocation to leaves (Fig. 2E), thereby increasing the R/S (Fig. 2F), whereas the phenotype of *SlTPP1* mutant plants was the opposite. Subsequently, we analyzed the R/S of plants of each genotype under normal conditions and found that overexpression of *SlTPP1* also increased the R/S, while knockout of *SlTPP1* led to a decrease in the R/S (Fig. 2G). In addition, under normal conditions, the soluble sugar content in roots of tomato plants of each genotype was consistent with the R/S: overexpression of *SlTPP1* increased soluble sugar content in roots, while the phenotype of *SlTPP1* knockout was the opposite (Fig. 2H). In summary, these results indicate that *SlTPP1* positively regulates drought tolerance in tomato by increasing the R/S and soluble sugar content in roots.

### *SlTPP1* overexpression positively regulates the expression of root cell wall synthesis genes.

**Figure 3:**
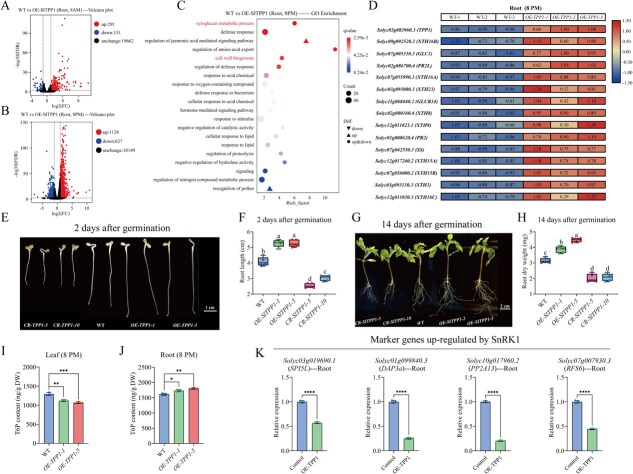
*SlTPP1* overexpression promotes the expression of root cell wall synthesis genes and root growth in tomato. (A, B) Analysis of differentially expressed genes in roots of WT and *OE-SlTPP1* tomato plants at 8 a.m. (A) and 8 p.m. (B). (C) GO enrichment (biological process) analysis of differentially expressed genes in roots of WT and *OE-SlTPP1* tomato plants at 8 p.m. (D) Analysis of cell wall synthesis gene expression in roots of WT and *OE-SlTPP1* tomato plants at 8 p.m. Gene expression values were normalized. (E, G) Phenotypic images of WT, *OE-TPP1*, and *CR-TPP1* tomato plants at 2 days (E) and 14 days (G) after seed germination. (E) Bar, 1 cm. (F) Bar, 2 cm. (F) Root length of WT, *OE-TPP1*, and *CR-TPP1* tomato plants at 2 days after seed germination. (H) Root DW of WT, *OE-TPP1*, and *CR-TPP1* tomato plants at 14 days after seed germination. (I, J) T6P content in leaves (I) and roots (J) of *OE-TPP1* tomato plants at 8 p.m. (K) Expression levels of SnRK1-upregulated marker genes in roots of *OE-TPP1* tomato plants. (F, H) Data are means ± SD (*n* = 5), and significance was analyzed by one-way ANOVA followed by Tukey’s test. Different lowercase letters indicate a significant difference (*P* < 0.05). (I–K) Data are means ± SD (*n* = 3). Statistical significance was analyzed using two-tailed Student’s *t* test (^*^P < 0.05, ^**^P < 0.01, ^***^P < 0.001, ^****^P < 0.0001).

To investigate the mechanism by which overexpression of *SlTPP1* increases the R/S in tomato, and considering the diurnal differences in plant sugar metabolism and sugar transport, we performed transcriptome analysis on the roots of WT and *OE-TPP1* tomato plants at two time points: 8 a.m. and 8 p.m. The results showed that the number of differentially expressed genes (DEGs) in *OE-TPP1* roots was much higher at 8 p.m. than at 8 a.m., indicating that overexpression of *SlTPP1* exerts a greater impact on roots at night (Fig. 3A and B). GO enrichment analysis revealed that the DEGs in roots at night were significantly enriched in the xyloglucan metabolic process and cell wall biosynthesis process (Fig. 3C). Further analysis demonstrated that overexpression of *SlTPP1* significantly upregulated the expression of genes related to root cell wall biosynthesis (Fig. 3D), suggesting that this might be one of the important reasons for promoting root growth and increasing the tomato R/S.

In addition, to further clarify the role of *SlTPP1* in regulating tomato root growth, we analyzed the root growth of tomato seeds germinated for 2 and 14 days, respectively. The results showed that 2 days after seed germination, compared with WT, the root length of *OE-TPP1* tomatoes was significantly increased, while that of *CR-TPP1* tomatoes was significantly decreased (Fig. 3E and F). Consistently, 14 days after seed germination, overexpression of *SlTPP1* significantly increased the DW of tomato roots, whereas the phenotype of *SlTPP1* knockout was the opposite. Taken together, *SlTPP1* can promote root growth by upregulating the expression of genes related to root cell wall biosynthesis in tomato at night, thereby increasing the R/S of tomato.

TPP catalyzes the conversion of T6P to trehalose [21]. To investigate whether *SlTPP1* overexpression affects tomato root growth by altering the T6P/SnRK1 signaling pathway, we analyzed the T6P content in leaves and roots of WT and *OE-TPP1* tomato plants. The results showed that at 8 p.m., *SlTPP1* overexpression significantly decreased T6P content in leaves but significantly increased that in roots (Fig. 3I and J). Furthermore, we analyzed the expression levels of SnRK1-upregulated marker genes in the roots of WT and *OE-TPP1* tomato plants. The results showed that the expression of these marker genes was significantly downregulated in the roots of *OE-TPP1* plants (Fig. 3K). Collectively, these findings suggest that *SlTPP1* overexpression may act by increasing T6P content in tomato roots and repressing the SnRK1 signaling pathway, thereby upregulating the expression of cell wall biosynthesis-related genes.

### Overexpression of *SlTPP1* inhibits the ethylene signal transduction pathway in tomato leaves.

**Figure 4:**
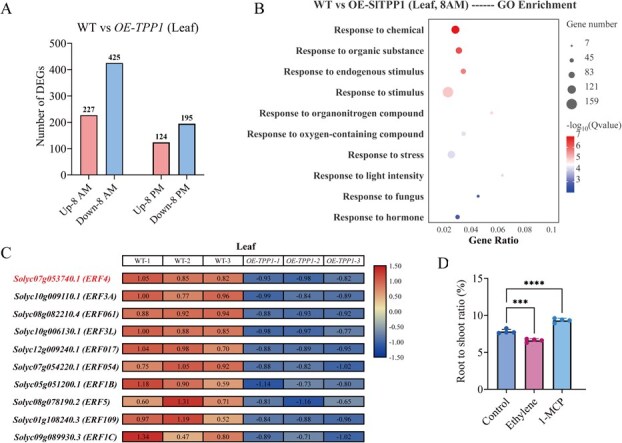
Overexpression of *SlTPP1* inhibits the ethylene signal transduction pathway in tomato leaves. (A) Analysis of differentially expressed genes in leaves of WT and *OE-TPP1* tomato plants at 8 a.m. and 8 p.m. (B) GO enrichment (Biological process) analysis of differentially expressed genes in roots of WT and *OE-TPP1* tomato plants at 8 a.m. (C) Gene expression analysis of ethylene signal transduction pathway in leaves of WT and *OE-TPP1* tomato plants at 8 a.m. Gene expression values were normalized. (D) The effect of foliar application of ethylene and the ethylene signaling inhibitor 1-MCP on the tomato R/S. Data are means ± SD (*n* = 4). Statistical significance was analyzed using two-tailed Student’s *t* test (^*^P < 0.05, ^**^P < 0.01, ^***^P < 0.001, ^****^P < 0.0001).

In addition to increasing the tomato R/S by promoting root growth, *SlTPP1* may also alter the R/S by affecting the aboveground parts, especially the leaves. Therefore, we also performed transcriptome analysis on the leaves of WT and *OE-TPP1* tomato plants at 8 a.m. and 8 p.m., and found that the number of DEGs between WT and *OE-TPP1* leaves was significantly higher at 8 a.m. than at 8 p.m. (Fig. 4A), indicating that *SlTPP1* overexpression exerts a more pronounced effect on tomato leaves in the morning. GO enrichment analysis of these DEGs revealed that genes related to responses to endogenous and exogenous stimuli, stresses, and hormones were significantly enriched (Fig. 4B). Further studies showed that the DEGs included many transcription factor genes involved in the ethylene signal transduction pathway, and all these genes were significantly downregulated in the leaves of *OE-TPP1* plants (Supplementary Fig. S2; Fig. 4C), indicating that overexpression of *SlTPP1* significantly inhibits the ethylene signal transduction pathway in tomato leaves. Previous studies have shown that activation or inhibition of the ethylene signal transduction pathway affects the plant R/S [41]. Therefore, to confirm the effect of the ethylene signal transduction pathway on the tomato R/S, we analyzed the changes in the R/S of tomato plants after treatment with ethylene and 1-methylcyclopropene (1-MCP) (Fig. 4D). The results showed that compared with the control, ethylene treatment decreased the tomato R/S, while 1-MCP treatment significantly increased it. These results indicate that *SlTPP1* can also increase the plant R/S by inhibiting the ethylene signal transduction pathway in tomato leaves.

### Transcription factor SlERF4 directly represses the expression of the *SlTPP1* gene.

**Figure 5:**
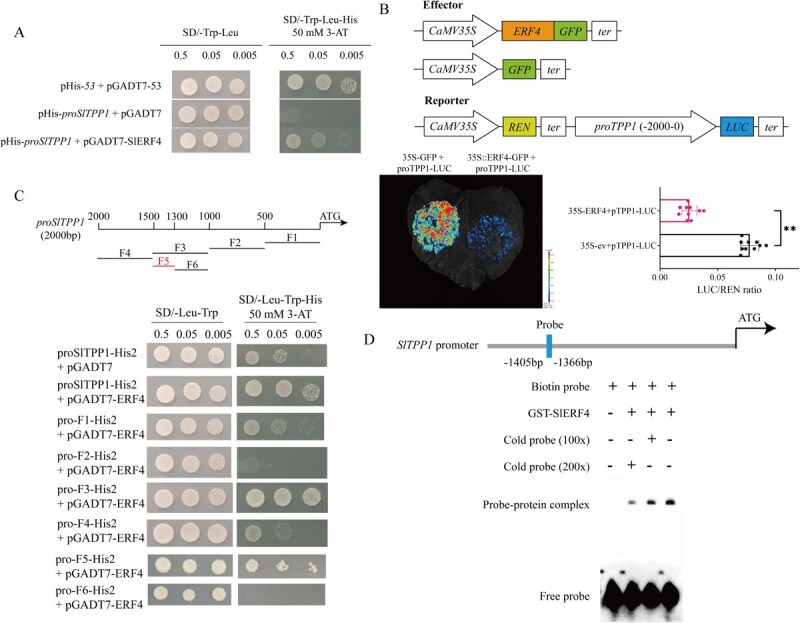
Transcription factor SlERF4 directly represses the expression of the *SlTPP1* gene. (A) Y1H assays identify that SlERF4 interacts with the promoter of *SlTPP1*. The combination of AD-53 and pHis-53 was used as a positive control, whereas the combination of AD and pHis-proSlTPP1 was used as a negative control. (B) Dual-luciferase assays indicate that SlERF4 inhibits the expression of *SlTPP1* in *N. benthamiana* leaves. Data are means ± SD (*n* = 10). Statistical significance was analyzed using two-tailed Student’s *t* test (^*^P < 0.05, ^**^P < 0.01, ^***^P < 0.001, ^****^P < 0.0001). (C) Promoter truncation combined with Y1H assay identifies the binding site of SlERF4 on the *SlTPP1* promoter. The promoter fragment F5 marked in red font is the interaction region. (D) EMSA assays demonstrate that SlERF4 can directly bind to the promoter of *SlTPP1 in vitro*. Competitors were used at 100- and 200-fold.

To investigate the molecular pathway by which *SlTPP1* gene expression responds to drought stress, we identified a transcription factor, SlERF4 (*Solyc07g053740*), that potentially interacts with the *SlTPP1* promoter through a yeast one-hybrid (Y1H) library screen. Results from the Y1H assay demonstrated that SlERF4 can bind to the *SlTPP1* promoter (Fig. 5A). Dual-luciferase assay results revealed that SlERF4 interacts with the *SlTPP1* promoter and inhibits its expression (Fig. 5B). To further identify the binding site of SlERF4 on the *SlTPP1* promoter, we truncated the *SlTPP1* promoter and analyzed the interaction using the Y1H system. The results indicated that SlERF4 directly interacts with the F5 fragment within the *SlTPP1* promoter region (Fig. 5C). According to previous reports [31], we identified a conserved ERF-binding element, the CE1 (Coupling Element 1) element, with the core sequence CACCG, in the F5 fragment. Subsequently, electrophoretic mobility shift assay (EMSA) experiments confirmed that SlERF4 can indeed directly bind to the CE1 element in the *SlTPP1* promoter region (Fig. 5D). Taken together, SlERF4 directly binds to and represses the expression of the *SlTPP1* gene.

### Expression pattern analysis of *SlERF4* and its response to drought.

**Figure 6:**
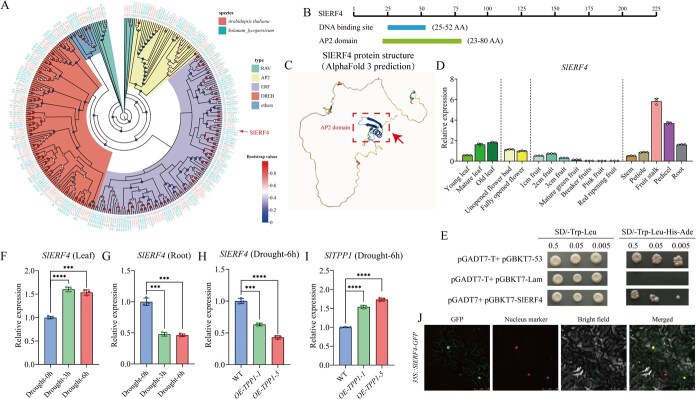
Expression pattern analysis of *SlERF4* and its response to drought. (A) Phylogenetic analysis of *TPP* homologs from *A. thaliana* and tomato (*S. lycopersicum*). Bootstrap support values (1000 replicates) are represented by circles of different colors. (B) Schematic diagram of the conserved domain of SlERF4 protein. (C) The structure of the SlERF4 protein predicted by AlphaFold 3. The red box encloses the AP2 domain. (D) Analysis of the temporal and spatial expression pattern of the *SlERF4* gene in tomato. Data are means ± SD (*n* = 3). (E) Validation of the transcriptional activation activity of SlERF4 by Y2H assay. The combination of AD-T and BD-53 was used as a positive control, whereas the combination of AD-T and BD-lam was used as a negative control. (F, G) qRT-PCR analysis of *SlERF4* expression dynamics in tomato leaves (F) and roots (G) at 3 and 6 h post-drought treatment. (H, I) Expression levels of *SlERF4* and *SlTPP1* genes in leaves of WT and *OE-TPP1* tomato plants at 6 h post-drought treatment. (F–I) Data are shown as means ± SD (*n* = 3). Statistical significance was analyzed using two-tailed Student’s *t* test (^*^P < 0.05, ^**^P < 0.01, ^***^P < 0.001, ^****^P < 0.0001). (J) Subcellular localization of tomato SlERF4-GFP fusion protein. Nucleus-mCherry is a nucleus marker with red fluorescence. Scale bars, 75 μm.

Prior to investigating the biological functions of *SlERF4*, we analyzed its expression pattern. The ERF transcription factor family is mainly classified into five types based on their domains and amino acid sequences [42]. Phylogenetic analysis revealed that SlERF4 belongs to the ERF-type ERF transcription factor (Fig. 6A). Analysis of the SlERF4 protein structure showed that amino acids 23–80 constitute a conserved AP2 domain (Fig. 6B). Subsequently, we predicted the protein structure of SlERF4 using AlphaFold3, and its AP2 domain is a key region for binding to the promoters of target genes (Fig. 6C). Results of temporal and spatial expression analysis indicated that *SlERF4* exhibits high expression in organs such as mature leaves, roots, pedicel, and fruit stalks (Fig. 6D). We then analyzed the transcriptional activation activity of SlERF4 using the yeast two-hybrid (Y2H) system, and found that yeast cotransformed with BD-SlERF4 and AD could grow on 4-deficient medium, demonstrating that SlERF4 has transcriptional activation activity (Fig. 6E). Furthermore, we analyzed the response of the *SlERF4* gene in tomato leaves and roots to drought using qRT-PCR. The results showed that, in contrast to the trend of *SlTPP1*, the *SlERF4* gene in leaves was upregulated after drought treatment (Fig. 6F), while that in roots was downregulated after drought treatment (Fig. 6G). Subcellular localization results indicated that SlERF4 is localized to the nucleus (Fig. 6H). In summary, SlERF4 is an ERF-type transcription factor localized in the nucleus, and it exhibits distinct expression responses to drought in tomato leaves and roots.

### *SlERF4* negatively regulates the R/S and drought tolerance in tomato plants

**Figure 7:**
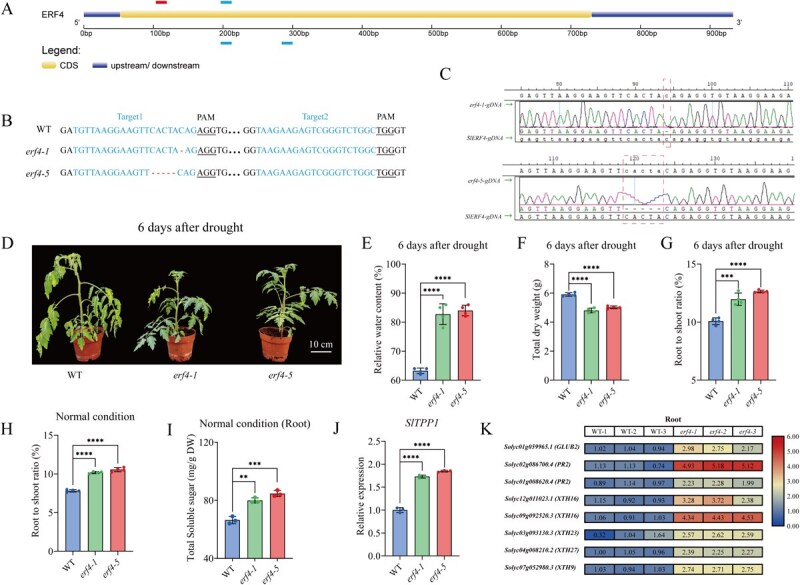
*SlERF4* negatively regulates the R/S and drought tolerance in tomato plants. (A) Schematic diagram showing the positions of *SlERF4* gene editing target sites in the DNA sequence. Red short horizontal lines denote the positions where mutations occurred in gene-edited plants. (B) Schematic diagram of the identification results of editing patterns in *SlERF4* gene-edited plants. Two mutants harbor deletions of 1 and 5 bp, respectively, at the first target site. (C) DNA sequencing chromatogram of the mutation site in *SlERF4* mutant plants. (D) Phenotypes of WT and *erf4* tomato seedlings after 6 days of drought treatment. Bar, 5 cm. (E–G) Analysis of relative leaf water content (E), total DW (F), and R/S (G) in WT and *erf4* tomato seedlings after 6 days of drought treatment. (H, I) Analysis of R/S (H) and total soluble sugar content in roots (I) in WT, and *erf4* tomato seedlings under normal conditions. (J) The expression level of the *SlTPP* gene in the roots of *erf4* mutant tomatoes. (K) qRT-PCR analysis of the expression of cell wall synthesis-related genes in the roots of *erf4* mutant tomatoes. The relative expression level of the gene in *erf4* mutants was calculated with the gene expression level in WT set as 1. (E–G, H–J) Data are means ± SD (*n* = 3 to 4). Statistical significance was analyzed using two-tailed Student’s *t* test (^*^P < 0.05, ^**^P < 0.01, ^***^P < 0.001, ^****^P < 0.0001).

To explore *SlERF4*’s role in regulating tomato R/S and drought tolerance, we generated two *SlERF4* mutants (*erf4-1* and *erf4-5*) via CRISPR/Cas9-mediated gene editing; these mutants carry 1- and 5-bp deletions in the coding sequence (CDS), respectively (Fig. 7A–C). After 3 days of drought treatment on WT and *erf4-1/5* tomato seedlings, we observed that WT tomato leaves exhibited obvious drought phenotypes, specifically leaf margin curling and mild wilting, whereas *erf4* mutant plants showed almost no drought phenotypes (Fig. 7D). Further analysis revealed that compared with WT, *erf4* mutant leaves had a higher RWC (Fig. 7E), indicating that *erf4* mutant plants possess enhanced drought tolerance. Furthermore, although the total DW of *erf4* mutants was lower than that of WT (Fig. 7F), their R/S was significantly higher than that of WT (Fig. 7G), suggesting that *SlERF4* can also regulate tomato drought tolerance by modulating R/S.

To further clarify the role of *SlERF4* in regulating R/S, we analyzed the R/S of WT and *erf4* tomato seedlings under normal conditions. The results showed that the R/S of *erf4* mutant tomatoes was also significantly higher than that of WT under normal conditions (Fig. 7I). In addition, the soluble sugar content in the roots of *erf4* mutant tomatoes was significantly higher than that in WT (Fig. 7J). To determine the regulatory effect of SlERF4 on *SlTPP1*, we analyzed the expression level of the *SlTPP1* gene in *erf4* mutant plants and found that *SlTPP1* expression was significantly upregulated in *erf4* mutants (Fig. 7K), indicating that SlERF4 indeed negatively regulates *SlTPP1* gene expression. To explore whether *SlERF4* also regulates R/S by affecting the expression of root cell wall synthesis-related genes, we analyzed the expression levels of these genes in the roots of WT and *erf4* mutant tomatoes. The results showed that the expression of cell wall synthesis-related genes in the roots of *erf4* mutants was significantly upregulated (Fig. 7L). In summary, *SlERF4* negatively regulates the R/S and drought tolerance in tomato plants.

### Drought-regulated ethylene biosynthesis mediates differential expression of *SlERF4* and *SlTPP1* in tomato roots and leaves

**Figure 8:**
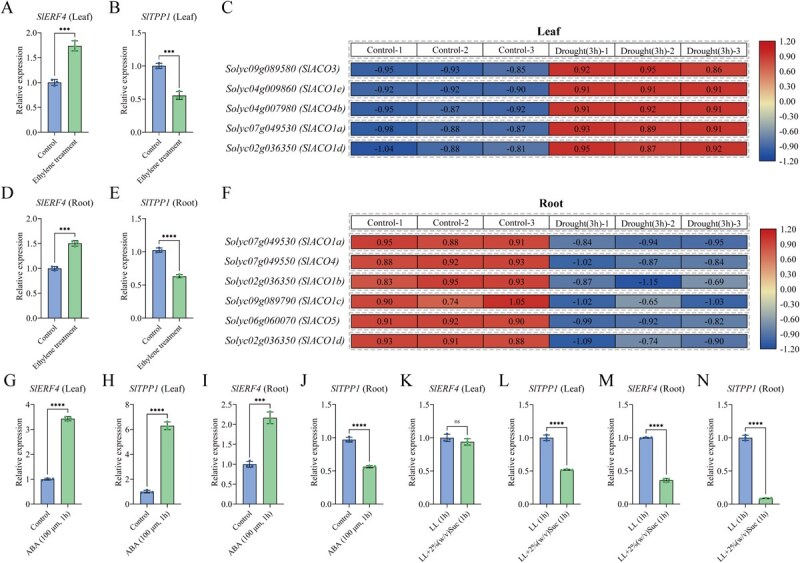
Drought-driven ethylene biosynthesis regulates differential expression of *SlERF4* and *SlTPP1* in tomato root and leaf tissues. (A, B) Effects of exogenous ethylene application on SlERF4 (A) and SlTPP1 (B) gene expression in tomato leaves. (C) Heatmap of *SlACOs* gene expression in tomato leaves after 3 h of drought treatment. Gene expression values were normalized. (D, E) Effects of exogenous ethylene application on *SlERF4* (D) and *SlTPP1* (E) gene expression in tomato leaves. (F) Heatmap of *SlACOs* gene expression in tomato roots after 3 h of drought treatment. Gene expression values were normalized. (G, H) Effects of exogenous ABA application on *SlERF4* (G) and *SlTPP1* (H) gene expression in tomato leaves. (I, J) Effects of exogenous ABA application on *SlERF4* (I) and *SlTPP1* (J) gene expression in tomato roots. (K, L) Effects of exogenous sucrose application on *SlERF4* (K) and *SlTPP1* (L) gene expression in tomato leaves. (M, N) Effects of exogenous sucrose application on *SlERF4* (M) and *SlTPP1* (N) gene expression in tomato roots. (A, B, D, E, G–N) Data are means ± SD (*n* = 3). Statistical significance was analyzed using two-tailed Student’s *t* test (^*^P < 0.05, ^**^P < 0.01, ^***^P < 0.001, ^****^P < 0.0001).

To investigate how drought treatment induces the differential expression of *SlERF4* and *SlTPP1* in tomato leaves and roots, we first analyzed the effects of ethylene treatment on the expression of these two genes in tomato leaves, given that *SlERF4* functions as an ethylene-responsive transcription factor. The results showed that exogenous ethylene application significantly upregulated the expression of the *SlERF4* gene (Fig. 8A and D) while markedly downregulating that of the *SlTPP1* gene (Fig. 8B and E) in tomato leaves and roots. Further analysis revealed that drought treatment significantly upregulated the expression levels of key ethylene biosynthesis genes, 1-aminocyclopropane-1-carboxylate oxidase (*SlACOs*), in tomato leaves (Fig. 8C), but concurrently downregulated *SlACOs* expression in roots (Fig. 8F), indicating that ethylene biosynthesis was enhanced in leaf tissues yet reduced in root tissues following drought exposure.

ABA and sugar signaling play crucial roles in plant responses to drought stress [43,44]. To investigate whether the differential expression of *SlERF4* and *SlTPP1* in tomato leaves and roots under drought treatment is regulated by ABA and sugar signaling, we analyzed the effects of exogenous ABA and sucrose treatments on the expression of these two genes in tomato leaves and roots. The results showed that in tomato leaves, exogenous ABA treatment significantly upregulated the expression of both *SlERF4* and *SlTPP1* genes (Fig. 8G and H). In contrast, in tomato roots, ABA treatment significantly upregulated *SlERF4* gene expression while concurrently downregulating that of *SlTPP1* (Fig. 8I and J). Exogenous sucrose had no significant effect on the expression of the *SlERF4* gene in tomato leaves, but significantly downregulated that of *SlTPP1* (Fig. 8K and L). In tomato roots, exogenous sucrose significantly downregulated the expression of both *SlERF4* and *SlTPP1* genes (Fig. 8M and N). Collectively, these results indicate that drought treatment regulates the expression of *SlERF4* and *SlTPP1* by modulating ethylene biosynthesis in tomato leaves and roots.

## Discussion

Global climate change has led to a decline in plants’ water recovery capacity, thus exploring the mechanisms to enhance plant drought tolerance holds global urgency [2]. Drought stress compromises plant productivity and development primarily by limiting water availability [3, 43, 44]. Trehalose-6-phosphate phosphatases (TPPs), which catalyze the conversion of trehalose-6-phosphate (T6P) to trehalose, are known to be crucial for drought tolerance [21]. Among the eight *TPP* genes identified in tomato, *SlTPP1* has been previously implicated in carbohydrate partitioning and growth promotion [22]. Our current study strengthens its specific role in drought adaptation. We demonstrated that drought stress significantly induced *SlTPP1* expression in roots, with no notable changes in other *TPPs* (Fig. 1A), suggesting a pivotal and specific function for *SlTPP1* in the root drought response. Conversely, *SlTPP1* expression was suppressed in leaves upon drought treatment (Fig. 1C), highlighting a contrasting tissue-specific regulatory pattern. Consistent with this, functional analyses using transgenic plants showed that *SlTPP1*-overexpressing lines exhibited higher leaf RWC under drought, while mutants were more susceptible (Fig. 2C), collectively indicating that *SlTPP1* acts as a positive regulator of drought tolerance in tomato.

Enhancing root growth and R/S to improve water acquisition is a key strategy for boosting drought tolerance in plants [16, 19]. In response to drought stress, plant roots upregulate metabolic activity to support water and nutrient uptake, thereby increasing the R/S [18]. Previous studies have demonstrated that sugar transport to the roots is an important mechanism for regulating root growth in plants. Drought and ABA activate SnRK2 kinases that phosphorylate sucrose transporters (e.g. SWEET11/12 in *Arabidopsis*), enhancing their activity and oligomerization to facilitate sucrose flux from shoots to roots [19]. Salt stress triggers PUB30-mediated degradation of HB24, suppressing *SWEET11* expression and inhibiting root growth [45]. In this study, to elucidate the mechanism by which *SlTPP1* increases the R/S in tomato, we conducted a transcriptome analysis of roots. We found that the impact of *SlTPP1* overexpression on differentially expressed genes was far greater during the night than in the morning, suggesting that *SlTPP1*-mediated adjustment of the R/S occurs primarily at night. This aligns with the documented diurnal variation in carbohydrate partitioning and root growth [22]. Furthermore, DEGs were significantly enriched and upregulated in pathways related to cell wall biosynthesis (Fig. 3D). The upregulation of these genes indicates promoted root growth in *SlTPP1*-overexpressing plants, a finding consistent with other reports linking cell wall remodeling to root development [46]. Since root growth demands substantial carbon skeletons and energy, we analyzed soluble sugar content and found it to be significantly higher in the roots of *SlTPP1* overexpressors. In summary, *SlTPP1* overexpression increases soluble sugar accumulation in roots, which in turn upregulates the expression of cell wall biosynthesis genes, thereby promoting root growth and increasing the R/S. This conclusion is further supported by the enhanced root growth phenotypes observed in *SlTPP1*-overexpressing lines at both 2 and 14 days after germination (Fig. 3E–H). Furthermore, we found that *SlTPP1* overexpression did reduce the T6P content in tomato leaves (Fig. 3I), which is consistent with its function of catalyzing the conversion of T6P to trehalose [47]. However, *SlTPP1* overexpression concurrently increased the T6P content in tomato roots (Fig. 3J) and repressed the expression of SnRK1-upregulated marker genes (Fig. 3K), indicating a positive correlation between root T6P content, the expression of cell wall biosynthesis-related genes, and root growth. Collectively, these results suggest that *SlTPP1* overexpression may be involved in regulating tomato root growth via the T6P-SnRK1 signaling pathway. Nevertheless, the specific molecular mechanisms and whether other signaling pathways are involved in modulating tomato root growth remain to be further elucidated.

Hormonal pathways are also key routes for regulating the root growth and R/S in plants. ABA accumulates rapidly in roots and leaves under stress, suppressing shoot growth by reducing cytokinins and altering auxin distribution, while ethylene modulation—exemplified in sorghum by *SbWRKY50* transcription factor repressing *ACS* genes to inhibit ethylene synthesis—promotes root expansion [12, 17]. Similarly, 1-MCP treatment increases root length in tomato plants [41]. Transcriptome analysis of tomato leaves revealed that overexpression of *SlTPP1* significantly downregulated the expression of genes involved in the ethylene signal transduction pathway (Fig. 4C). To clarify the effect of the ethylene signaling pathway on the R/S of tomato plants, we treated tomato seedlings with ethylene and 1-MCP. Consistent with the findings in *Arabidopsis thaliana*, exogenous ethylene application reduced the R/S of tomato plants, whereas exogenous 1-MCP application, which inhibits ethylene signal transduction, increased the R/S of tomato plants (Fig. 4D). These results indicate that overexpression of *SlTPP1* can increase the R/S of plants by inhibiting the ethylene signal transduction pathway in leaves. However, whether *SlTPP1* is involved in ethylene signaling regulation through the classical T6P-SnRK1 pathway requires further investigation in future studies.

As an enzyme-encoding gene, the response of *SlTPP1* to drought is regulated by upstream transcription factors. The transcription factor-*TPP* module has been reported in numerous aspects of regulating plant growth and development as well as stress responses. For example, the sugar-induced rice NAC transcription factor OsNAC23 can directly repress the transcription of *OsTPP1*, thereby simultaneously increasing the level of T6P and decreasing the level of trehalose, and further promoting the allocation of carbon from source organs to sink organs [48]. To identify the upstream transcription factors of *SlTPP1*, we performed Y1H library screening and identified an ERF transcription factor, SlERF4. Furthermore, we further verified through Y1H assays, dual-luciferase reporter assays, and EMSA that SlERF4 can directly bind to the promoter of *SlTPP1* and repress its expression (Fig. 5). In *SlERF4* mutant plants, we detected that the expression level of *SlTPP1* was upregulated (Fig. 7K), which was consistent with the results of the dual-luciferase reporter assay. Furthermore, we found that the expression pattern of the *SlERF4* gene in tomato leaves and roots after drought treatment was exactly the opposite of that of *SlTPP1* (Fig. 6F and G), which also reflected the repressive effect of SlERF4 on *SlTPP1* expression, indicating that SlERF4 is likely a key upstream regulatory factor of *SlTPP1* in response to drought stress. Results of functional validation indicated that *SlERF4* negatively regulates the R/S and drought tolerance of tomato plants (Fig. 7D–G).

AP2/ERF transcription factors are crucial for enhancing plant drought tolerance via various mechanisms. They improve WUE and photosynthesis by regulating stomatal conductance and carbon assimilation [49, 50]. Additionally, they promote osmotic adjustment through the accumulation of proline and soluble sugars [37]. These factors also integrate hormone signaling pathways, including ABA, JA, and ethylene, to coordinate stress responses [30, 33]. Furthermore, they maintain ROS homeostasis by activating antioxidant enzymes [35], regulate cuticular wax biosynthesis to minimize water loss [34], and participate in epigenetic modifications like histone demethylation to fine-tune gene expression networks [51]. These mechanisms collectively establish plants to adapt to water scarcity by balancing growth and defense strategies. In addition, emerging evidence suggests that certain ERFs, for example, CmERF053 in chrysanthemum, can enhance drought tolerance by promoting lateral root formation [52]. In our study, SlERF4 acts as an upstream regulator of *SlTPP1*. To investigate whether *SlERF4* regulates tomato root growth and R/S through the same pathway as *SlTPP1*, we analyzed the soluble sugar content and the expression of cell wall synthesis-related genes in the roots of *SlERF4* mutant tomato plants. Similar to the results of *SlTPP1*, knockout of *SlERF4* significantly increased the soluble sugar content in tomato roots as well as the expression of key genes involved in cell wall synthesis (Fig. 7K). These results indicate that the SlERF4–SlTPP1 is an important module that regulates the R/S of tomato and thereby affects plant drought tolerance.

Under drought conditions, *SlTPP1* and *SlERF4* exhibited opposite expression patterns in tomato leaves and roots, which is consistent with the inhibitory effect of SlERF4 on *SlTPP1* expression. However, what signaling pathway accounts for the differential expression of these two genes in leaves and roots under drought stress? ABA is the primary hormone mediating plant responses to drought stress [53]. We analyzed the effects of ABA on the expression of *SlTPP1* and *SlERF4* in tomato leaves and roots, and found that ABA treatment significantly upregulated the transcript levels of both *SlERF4* and *SlTPP1* in leaves (Fig. 8G and H). These results indicate that ABA is not the signal molecule responsible for the differential expression of *SlTPP1* and *SlERF4* between leaves and roots. Soluble sugars also play critical roles in plant adaptation to drought stress [4]. Exogenous sucrose treatment assay showed that sucrose application remarkably downregulated the expression of both *SlERF4* and *SlTPP1* in roots (Fig. 8M and N), suggesting that soluble sugars are also not the signaling molecules causing the differential expression of *SlERF4* and *SlTPP1* in leaves and roots under drought conditions. However, previous studies have demonstrated that drought activates the kinase activity of SnRK2 via ABA signaling, which elevates the phosphorylation levels of *Arabidopsis* sucrose transporters SWEET11 and SWEET12 to enhance their sugar transport capacity, thereby promoting the translocation of sucrose from aboveground shoots to roots [19]. Our findings revealed that exogenous sucrose application represses the expression of *SlERF4* in tomato roots. Accordingly, sugar transport triggered by drought may partially contribute to the regulation of *SlERF4* expression in tomato roots under drought stress.

Considering that *SlERF4* is an ethylene-responsive transcription factor, we hypothesized that differential ethylene signaling between tomato leaves and roots under drought stress might lead to the divergent expression of *SlTPP1* and *SlERF4*. The results showed that exogenous ethylene application indeed upregulated *SlERF4* expression while downregulating *SlTPP1* expression in both leaves and roots (Fig. 8). Furthermore, we found that drought treatment significantly induced the expression of *SlACOs* genes, which are key regulators of ethylene biosynthesis, in leaves, whereas their expression was markedly repressed in roots (Fig. 8C and F). This expression pattern of *SlACOs* was consistent with the differential expression profiles of *SlERF4* and *SlTPP1* in leaves and roots under drought stress. Collectively, these findings demonstrate that the differential ethylene signaling between leaves and roots is the major signal regulating the divergent expression of *SlERF4* and *SlTPP1* in tomato under drought conditions.

In addition, overexpression of *SlTPP1* represses the expression of genes involved in the ethylene signal transduction pathway in leaves, including the transcription factor *SlERF4* (Fig. 4C). In turn, SlERF4 can directly bind to and repress the expression of *SlTPP1*. Thus, a SlTPP1–SlERF4–SlTPP1 positive feedback regulatory loop is formed in the leaves of *SlTPP1*-overexpressing tomato plants, which maintains the expression of *SlTPP1* at a high level in these leaves. This consequently facilitates the synthesis of more sucrose in leaves and the transport of sucrose to sink organs such as roots, a finding that has been reported in previous studies [22].

**Figure 9:**
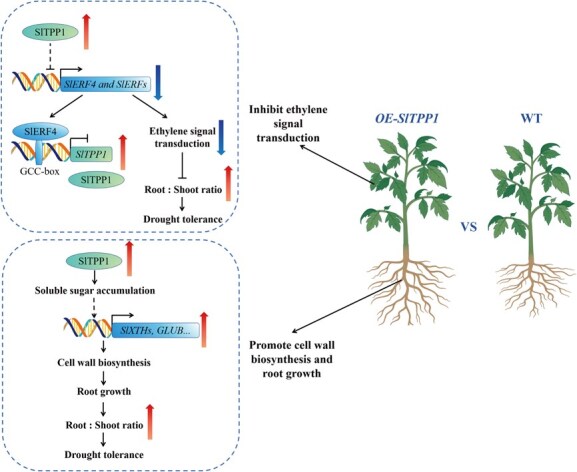
A proposed model of *SlTPP1* enhances drought tolerance by modulating the root/shoot ratio (R/S) in tomato. On one hand, overexpressing *SlTPP1* increases the soluble sugar content and indirectly upregulates the expression of cell wall biosynthesis genes in roots to promote root growth. On the other hand, *SlTPP1* represses the expression of genes (including *SlERF4*) involved in the ethylene signal transduction pathway in leaves, thus increasing the R/S of tomato plants. Furthermore, transcription factor SlERF4 can directly bind to and repress the expression of *SlTPP1*, thereby forming a positive feedback loop that promotes the expression of *SlTPP1* in the leaves of *SlTPP1*-overexpressing tomato plants.

Based on our findings, we have proposed a working model for the mechanism by which *SlTPP1* regulates drought tolerance in tomato plants (Fig. 9). Compared with WT, on one hand, overexpressing *SlTPP1* increases the soluble sugar content and indirectly upregulates the expression of cell wall biosynthesis genes in roots to promote root growth; on the other hand, *SlTPP1* represses the expression of genes (including *SlERF4*) involved in the ethylene signal transduction pathway in leaves, thus increasing the R/S of tomato plants. Furthermore, transcription factor SlERF4 can directly bind to and repress the expression of *SlTPP1*, thereby forming a positive feedback loop that promotes the expression of *SlTPP1* in the leaves of *SlTPP1*-overexpressing tomato plants. Collectively, our results elucidate a novel SlERF4–SlTPP1 regulatory module which enhances drought tolerance in tomato plants by increasing R/S.

## Materials and methods

### Plant materials and growth conditions

The wild-type (WT) tomato (*S. lycopersicum* L. cv Moneymaker) and transgenic lines within this genetic background were cultivated in a glasshouse using standard pots filled with commercial substrate. The plants were maintained at 16°C/26°C (night/day), with a photoperiod of 12 h and a photosynthetic photon flux density (PPFD) of approximately 350 μmol m^−2^ s^−1^. The *SlTPP1*-overexpressing tomato lines (*OE-TPP1-1/5*), *SlTPP1* knockout tomato lines (*CR-TPP1-5/10*), and proSlTPP1-GUS tomato plants have been obtained in our previously published article [22]. The generation of SlERF4 (*erf4-*1/5) knockout mutants were conducted via the CRISPR/Cas9 method. *Nicotiana benthamiana* were grown in an artificial chamber at 16°C/26°C (night/day) with 16-h light/8-h dark photoperiod and PPFD of 350 μmol m^−2^ s^−1^.

### Plasmid construction and plant transformation

To obtain the CRISPR/Cas9 vector of SlERF4, the target sequences were designed according to the online tool CRISPR-P 2.0 (http://crispr.hzau.edu.cn/CRISPR2/) [54]. The primers were designed and used to amplify the three fragments (containing target sequences) according to the method as previously reported [55]. Then the three fragments were cloned into a pTX041 at the BsaI sites by PCR reaction [56]. The CRISPR/Cas9 vector was validated by sequencing, and then was introduced into *Agrobacterium tumefaciens* strain EHA105 using the chemical transformation method and finally transformed into Moneymaker (MM) using leaf disc transformation method [57]. The identification of *erf4* mutants (T0 generation) were genotyped through sequencing the fragments containing target sites, which were amplified by specific primers (Table S1). The T0 homozygous mutant seeds (T1 generation) of *erf4* were selected and selfed to obtain T2 generation mutants without the Cas9 marker, which were used for subsequent experiments. For subcellular localization of SlERF4, the full-length CDS of SlERF4 was amplified with the specific primers (Table S1) and then cloned into pCAMBIA1300-35S-EGFP (EcoRI and BamHI) vector.

### Drought treatment

For proSlTPP1-GUS tomato plants used for GUS staining, 7 days after seed germination, 15% (w/v) PEG6000 was applied to plug trays by watering to simulate drought stress. At 0 and 3 h after treatment, root systems were harvested, rinsed thoroughly, and immediately subjected to GUS staining. Fourteen days after seed germination, the same drought stress simulation was performed by watering plug trays with 15% (w/v) PEG6000. At 0 and 3 h after treatment, functional leaves were sampled and immediately used for GUS staining.

For gene expression analysis in tomato leaves and roots after drought treatment, 14-day-old WT tomato plants were watered with 15% (w/v) PEG6000 to simulate drought stress. At 0, 3, and 6 h after treatment, leaves and roots were sampled separately, and immediately frozen in liquid nitrogen until subsequent analysis.

For the functional verification experiment of *SlTPP1* regulating tomato drought tolerance, 5-week-old WT, *OE-TPP1*, and *CR-TPP1* tomato plants were watered with 15% (w/v) PEG6000 to simulate drought stress for 6 days. Subsequently, plant phenotypes, relative leaf water content, plant DW, and its distribution were analyzed. In addition, the aforementioned plants were simultaneously cultivated under normal growth conditions, with their R/S and soluble sugar content in roots determined. For transcriptome analysis, samples of mature leaves (at 8 a.m.) and roots (at 8 a.m. and 8 p.m.) were collected from WT and *OE-TPP1* tomato plants grown under normal conditions.

For the functional verification experiment of *SlERF4* regulating tomato drought tolerance, 5-week-old WT and *erf4* mutant tomato plants were watered with 15% (w/v) PEG6000 to simulate drought stress for 6 days. Subsequently, plant phenotypes, relative leaf water content, plant DW, and its distribution were analyzed. In addition, the aforementioned plants were simultaneously cultivated under normal growth conditions, with their R/S and soluble sugar content in roots determined. For gene expression analysis, root samples were collected from WT and *erf4* mutant tomato plants grown under normal conditions.

### Ethylene, ABA, and sugar treatments

To investigate the effects of ethylene on the expression of *SlERF4* and *SlTPP1* in tomato leaves and roots, 3-week-old tomato seedlings were subjected to foliar spray with 200 μl/l ethephon, while seedlings sprayed with distilled water served as the control. After 3 h of treatment, leaf and root samples were collected for the analysis of *SlERF4* and *SlTPP1* gene expression levels.

To examine the impacts of ABA and sugar on the expression of *SlERF4* and *SlTPP1* in tomato leaves and roots, the root systems of 3-week-old tomato seedlings were thoroughly rinsed and then transferred to glass vials filled with distilled water for hydroponic culture. Subsequently, ABA or sucrose was added to the hydroponic solution to reach final concentrations of 100 μM and 2% (w/v), respectively; seedlings cultured in distilled water alone were set as the control. Seedlings in the ABA treatment and control groups were maintained under normal light conditions, whereas those in the sucrose treatment and control groups were cultured in dark conditions to avoid the adverse effects of excessive foliar sugar accumulation on plant growth. After 3 h of treatment, leaf and root samples were harvested to determine the expression levels of *SlERF4* and *SlTPP1*.

### Relative water content

Tomato mature leaves were used for leaf RWC measurements. The fresh weight (FW) was immediately recorded using a composite sample of fresh leaves. The leaves were submerged in water for 6 h to measure their turgid weight. Then, the leaves were oven-dried at 105°C for 30 min to deactivate enzymes, followed by drying at 80°C for approximately 48 h until constant weight, and the result was recorded as DW. The RWC was calculated as follows: RWC = (FW – DW)/(turgid weight – DW) × 100%.

### RNA extraction, cDNA synthesis, and qRT-PCR

The mature leaf and root samples were ground into fine powder using liquid nitrogen. Total RNA extraction, detection, cDNA synthesis, and quantitative real-time RT-PCR analysis were conducted following previously established methodologies [58]. The resulting cDNA products were diluted to a concentration of 100 ng/μl and employed as templates for qRT-PCR. The specific primer pairs utilized for the qRT-PCR analysis are listed in Table S1. Gene expression analysis was performed in three biological replicates for each sample. The relative gene expressions were assessed utilizing *SlActin7* (*Solyc03g078400*) as the internal control and were calculated using the 2^−ΔΔCt^ method [59]. Subsequent data analysis was carried out employing Excel 2019 software, and statistical analysis was performed using SPSS 23.0 software. Graphs were generated using Prism 10.4. Gene expression heatmaps were plotted using TBtools 2 [60].

### GUS staining

Tomato leaves and roots of proSlTPP1-GUS transgenic tomato plants were used for GUS staining. GUS assays were carried out as previously reported [61]. Briefly, samples were first immersed in 90% (v/v) acetone on ice for 15 min and then transferred to GUS staining solution at 37°C for 2–12 h. Then samples were transferred to 70% ethanol to stop staining, and distaining solvent (ethanol/acetic acid, 6:1 in volume) was used to remove pigments. The GUS-stained tissues were photographed using a Sony digital camera (NEX7).

### Measurement of DW, DW percentage, and R/S ratio

Tomato plants were destructively harvested after drought treatment or under normal conditions. Each plant was separated into roots, stems, and leaves. Samples were heated for 30 min at 105°C and then dried to constant weight at 65°C. The leaf mass fraction, stem mass fraction, and root mass fraction are equal to the DW of leaves, stems, and roots divided by the total DW of the plant. The R/S was calculated by the following equation:


$$ \mathrm{R}/\mathrm{S}\ \left(\%\right)=\mathrm{Root}\ \mathrm{DW}/\left(\mathrm{Total}\ \mathrm{DW}\hbox{--} \mathrm{R}\mathrm{oot}\ \mathrm{DW}\right)\ast 100. $$

### Quantification of total soluble sugar and T6P content

The quantification of total soluble sugar in roots followed a previously reported method with slight adaptations [62]. Briefly, freeze-dried roots were ground into powder using a high-throughput tissue grinder, and then were mixed with 4 ml of 80% (v/v) ethanol and incubated at 80°C for 30 min. After centrifugation at 8000 rpm for 10 min, the supernatants were collected, and this extraction process was repeated two times. The supernatant was brought to a final volume of 50 ml with distilled water, and then the total soluble sugar content was determined using the anthrone-sulfuric acid method. The quantification of T6P content in tomato leaves and roots were performed as previously reported [63].

### RNA sequencing

Total RNA extracted from samples of leaves and roots was used to construct the cDNA libraries. RNA sequencing was sequenced on the Illumina sequencing platform by Genedenovo Biotechnology Co., Ltd (Guangzhou, China). Gene expression levels were estimated by fragments per kilobase of transcript per million fragments (FPKM). Differentially expressed genes (DEGs) were identified using DESeq2. Genes with an adjusted *P* value <0.01 and log2 ratio >2 found by DESeq2 were assigned as differentially expressed. The DEGs were selected for Gene Ontology (GO) enrichment analysis focusing on biological processes.

### Y1H assay

The 2000-bp promoter sequence upstream of the ATG start codon of *SlTPP1* was cloned into the pHis2 vector. The primers utilized for these cloning processes are detailed in Table S1. The pGADT7-SlERF4 and pHis2-proSlTPP1 plasmids were cotransformed into yeast strain Y187. Transformed yeast cells were grown on synthetic dextrose (SD)/-Leu-Trp and SD/-Leu-Trp-His with 3-AT (50 mM) solid medium. The plates were incubated at 28°C for 3–4 days, and yeast growth conditions were used to access the interaction between the protein and promoters. pHis2-proSlTPP1/pGADT7 was used as the negative control. Yeast transformation and medium preparation were performed according to the instructions provided by Clontech.

### Ethylene and 1-MCP treatments

To investigate the effect of ethylene signaling on the R/S of tomato seedlings, 4-week-old WT tomato plants were placed in sealable 1-m^3^ growth chambers. For the ethylene spraying treatment, 210 mg of ethephon (IE3470; Solarbio, Beijing, China) was dissolved in 1 l of distilled water to a final concentration of 200 μl/l, with 0.05% Tween 20 added as a surfactant. The ethephon solution was uniformly sprayed onto tomato leaves until droplets dripped off, and distilled water spraying served as the control. For the 1-MCP treatment, 100 mg of 1-MCP powder (CM7233; Huayueyang, Beijing, China) was dissolved in 100 ml of water, and the treatment was maintained for 4 h. Subsequently, tomato plants were transferred to a greenhouse for further growth. After 10 days, plant dry matter distribution was determined.

### Dual-luciferase reporter assay

Dual-luciferase transient transcriptional activity assay was performed as previously described [64]. The 2000-bp promoter sequence upstream of the ATG start codon of *SlTPP1* was cloned into the pGreen II-0800-Luc vector as reporter constructs. 35S::SlERF4-GFP and 35S-GFP were as effector constructs. The primers utilized for these cloning processes are detailed in Table S1. 35S-GFP/proSlTPP1-LUC and 35S::SlERF4-GFP/proSlTPP1-LUC separately were cotransformed into the leaves of *N. benthamiana*. On the first day postinoculation, the samples were subjected to dark treatment, and on the second day, they were transferred to normal light conditions for subsequent cultivation. After expression in leaves for 48 h, the tobacco leaf samples from the infected areas were collected and LUC and REN activities were measured by the Dual Luciferase Reporter Gene Assay Kit (11402ES60; YEASEN, Shanghai, China). The relative activity of promoters was calculated by the LUC/REN ratio and detected by luminometer (Promega, GloMax2020). For the infected regions of the remaining tobacco leaves, a solution of D-luciferin potassium salt (1 mM) was injected from the abaxial side. After being placed at room temperature in the dark for 3–5 min, the leaves were detected and imaged using a plant *in vivo* imaging system (VILBER, Fusion FX7.Edge Spectra). A rainbow-colored pseudocolor was applied to the images to analyze the intensity of promoter activity.

### Electrophoretic mobility shift assay

EMSA was performed as previously described [65]. Based on the sequences of potential interacting promoter elements and their flanking sequences, sequences with a length of approximately 30–40 bp (including the forward sequence and the reverse complementary sequence) were designed as probes, and the 5′ end of the probes was labeled with biotin. Unlabeled probes served as competing cold probes. The probes utilized in this assay are detailed in Table S1. The full-length CDS of SlERF4 was cloned into the pGEX-4T-1 vector by using Uniclone One Step Seamless Cloning Kit (SC612; Genesand, Beijing, China). Primers used for plasmid construction were shown in Table S1. Recombinant GST-SlERF4 plasmid was transformed into *Escherichia coli* competent cells BL21 for prokaryotic expression. The expression was induced with 0.2 mM IPTG at 25°C for 6 h. Bacterial pellets were collected by centrifugation and used for protein purification. GST-SlERF4 protein was purified using a GST protein purification kit (SM002005; smart-lifesciences, Changzhou, China), following the manufacturer’s instructions. The recombinant GST-SlERF4 protein was incubated with biotin-labeled probes and competition probes, using a LightShift Chemiluminescent EMSA kit (Thermo Fisher, 20 148), at 25°C for 20 min. The binding reactions were terminated by adding native sample loading buffer, and the samples were separated on a native-PAGE gel prepared using the EMSA PAGE Gel Preparation Kit (MH1041; Coolaber, Beijing, China). The binding signal was detected by chemiluminescence image-analysis system (Tanon-5200; Tanon, Shanghai, China).

### Phylogenetic analysis and protein structure prediction

Protein sequences of ERFs in *A. thaliana* and *S. lycopersicum* were obtained from the National Center for Biotechnology Information (NCBI) database (https://www.ncbi.nlm.nih.gov/) and aligned with MUSCLE. The phylogenetic tree was inferred based on the Maximum Likelihood method with 1000 bootstrap replicates using MEGA 11 software [66]. The online software TVBOT (https://www.chiplot.online/tvbot.html) was used to beautify the phylogenetic tree [67]. The protein structure of SlERF4 was predicted using AlphaFold3 (https://alphafoldserver.com/).

### Spatiotemporal expression analysis of the *SlERF4* gene

For the spatiotemporal expression pattern analysis of *SlERF4*, various organs and tissues at different growth stages of MM tomato plants were sampled, and their RNA was extracted for detection of *SlERF4* expression by qRT-PCR.

### Analysis of transcriptional activation activity

The transcriptional activation activity of SlERF4 was analyzed using the Y2H system. Y2H assays were performed according to the instructions provided by Clontech. The full-length CDS of SlERF4 was cloned into the pGBKT7 vector. The primers utilized for this cloning process are detailed in Table S1. The pGADT7 and pGBKT7-SlERF4 vectors were cotransformed into yeast strain Y2HGold. Transformed yeast cells were grown on synthetic dextrose (SD)/-Leu-Trp (DDO) and SD/-Leu-Trp-His-Ade (QDO) with X-α-gal (40 μg ml^−1^) solid medium. The plates were incubated at 28°C for 3–4 days, and yeast growth conditions were used to access the interaction between the proteins. pGBKT7-P53/pGADT7-T was used as the positive control, while pGBKT7-Lam/pGADT7-T was used as the negative control.

### Subcellular localization

The subcellular localization vector 35S::SlERF4-EGFP was introduced into *A. tumefaciens* strain *EHA105* and then cotransformed into the leaves of 4-week-old *N. benthamiana* plants together with RNA silencing suppressor P19. The injected tobacco plants were first kept in dark conditions for 1 day, followed by 1 day of normal light cultivation, after which they were observed and photographed using a laser scanning confocal microscope (Leica, TCS SP8).

### Statistical analysis

The experimental design involved three biological replicates, where each biological replicate was evaluated through three independent technical replicates for the testing and subsequent analysis of experimental results. Statistical analysis was performed using SPSS 23.0 and GraphPad Prism version 10.4. Unpaired Student’s *t* test (two-tailed) was used for significant difference analysis between two groups. One-way ANOVA followed by Tukey’s multiple comparisons test was used for significant difference analysis among multiple samples. Asterisks indicate significant differences (^*^P < 0.05, ^**^P < 0.01, ^***^P < 0.001, and ^****^P < 0.0001). Different lowercase letters indicate significant differences (*P* < 0.05).

### Accession numbers

Genes associated with this study are accessible through the Sol Genomics Network database (https://solgenomics.sgn.cornell.edu/). Table S2 contains the accession numbers of this article mentioned.

## Supplementary Material

Web_Material_uhag070

## Data Availability

RNA sequencing data were deposited to SRA (https://www.ncbi.nlm.nih.gov/sra) with BioProject accession number PRJNA1366651 (root) and PRJNA1367885 (leaf). The data underlying this article are available in the article and in its online supplementary material.
