# Supplementary material for: A SlERF4–SlTPP1 module enhances drought tolerance in tomato by increasing the root/shoot ratio
Source: Hortic Res. 2026 Mar 2;13(6):uhag070. doi: 10.1093/hr/uhag070 (PMC13253350; doi:10.1093/hr/uhag070)
Supplement: Web_Material_uhag070 [file web_material_uhag070.zip › Fig. 1 - Fig. 9.pdf]

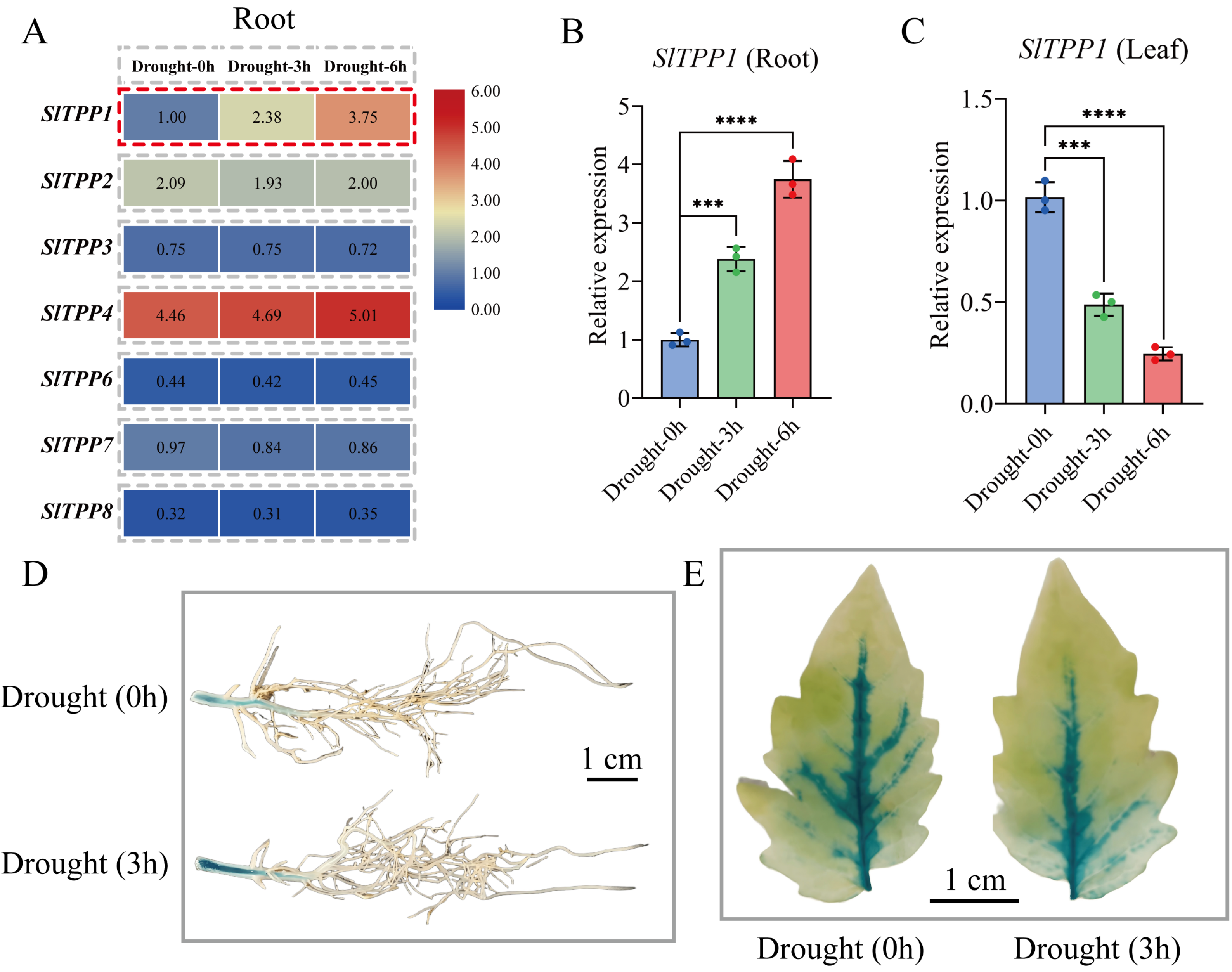
A
Root
Drought-0h
Drought-3h
Drought-6h
SlTPP1
1.00
2.38
3.75
SlTPP2
2.09
1.93
2.00
SlTPP3
0.75
0.75
0.72
SlTPP4
4.46
4.69
5.01
SlTPP6
0.44
0.42
0.45
SlTPP7
0.97
0.84
0.86
SlTPP8
0.32
0.31
0.35
6.00
5.00
4.00
3.00
2.00
1.00
0.00
B
SlTPP1 (Root)
Relative expression
****
***
C
SlTPP1 (Leaf)
Relative expression
****
***
D
Drought (0h)
Drought (3h)
1 cm
E
1 cm
Drought (0h)
Drought (3h)

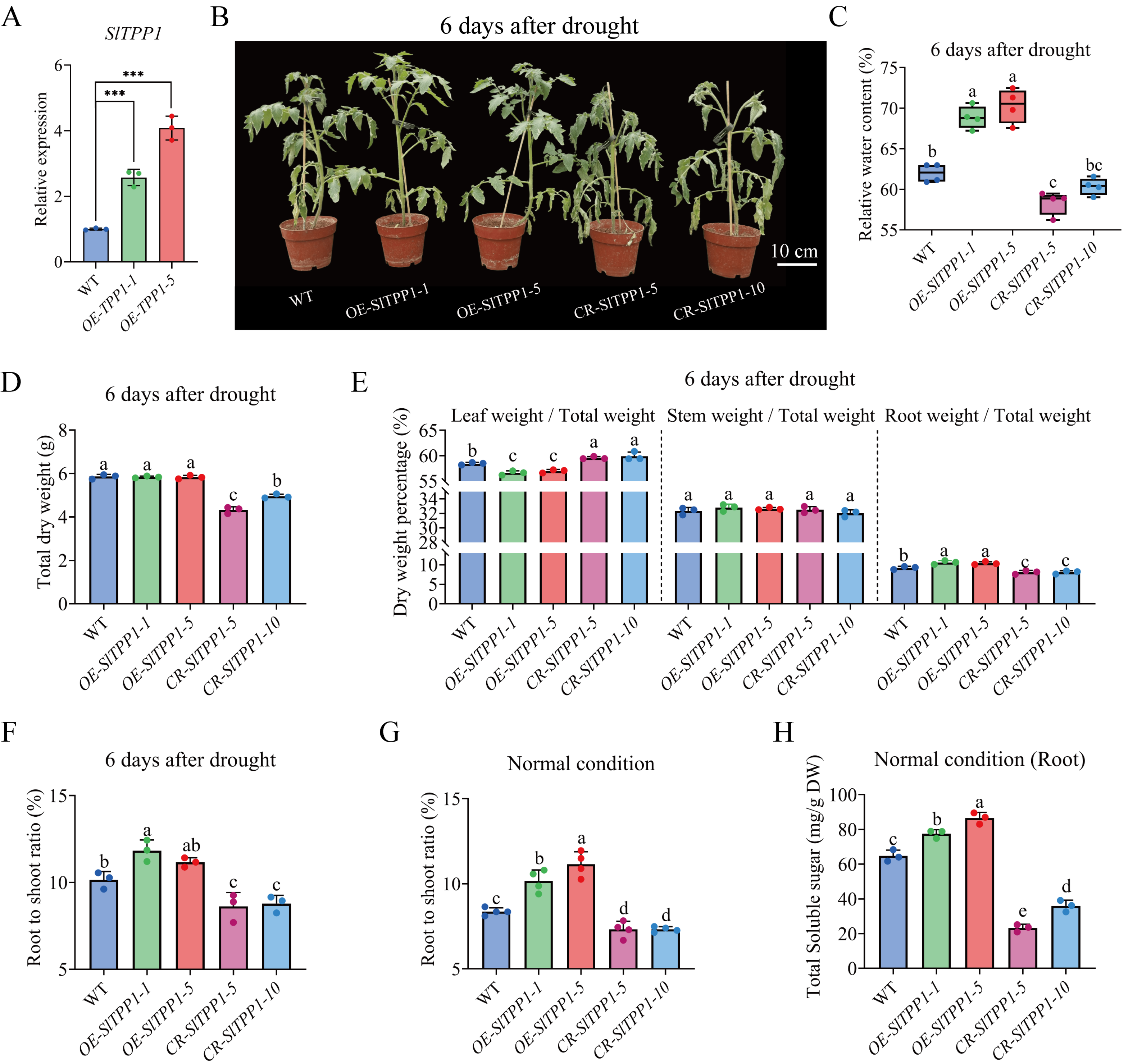
A
SlTPP1
Relative expression
***
***
WT
OE-TPP1-1
OE-TPP1-5
B
6 days after drought
10 cm
WT
OE-SlTPP1-1
OE-SlTPP1-5
CR-SlTPP1-5
CR-SlTPP1-10
C
6 days after drought
Relative water content (%)
D
6 days after drought
Total dry weight (g)
E
6 days after drought
Leaf weight / Total weight
Stem weight / Total weight
Root weight / Total weight
Dry weight percentage (%)
F
6 days after drought
Root to shoot ratio (%)
G
Normal condition
Root to shoot ratio (%)
H
Normal condition (Root)
Total Soluble sugar (mg/g DW)

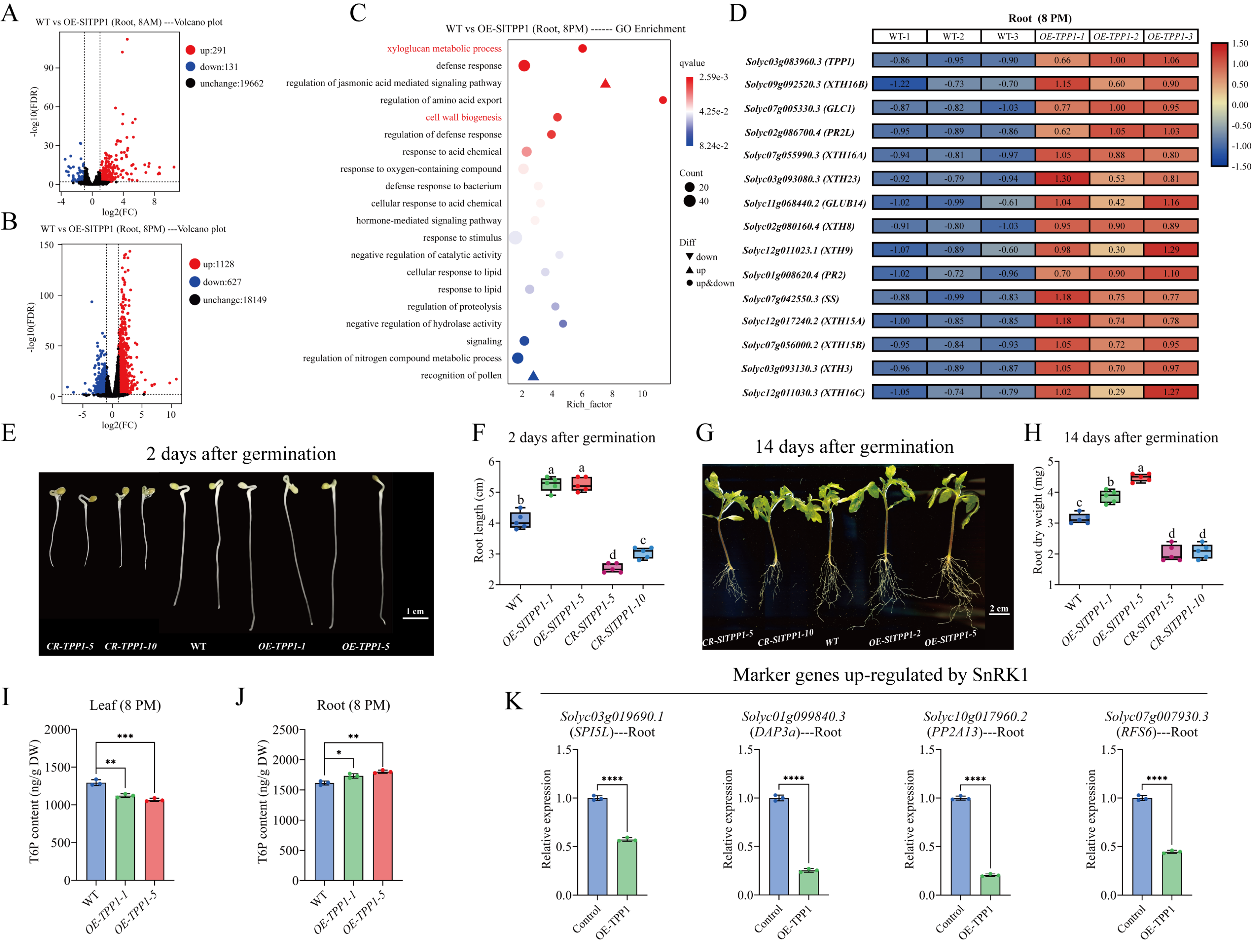
A
WT vs OE-SlTPP1 (Root, 8AM) ---Volcano plot
up:291
down:131
unchange:19662
-log10(FDR)
log2(FC)
B
WT vs OE-SlTPP1 (Root, 8PM) ---Volcano plot
up:1128
down:627
unchange:18149
C
WT vs OE-SlTPP1 (Root, 8PM) ------ GO Enrichment
xyloglucan metabolic process
defense response
regulation of jasmonic acid mediated signaling pathway
regulation of amino acid export
cell wall biogenesis
regulation of defense response
response to acid chemical
response to oxygen-containing compound
defense response to bacterium
cellular response to acid chemical
hormone-mediated signaling pathway
response to stimulus
negative regulation of catalytic activity
cellular response to lipid
response to lipid
regulation of proteolysis
negative regulation of hydrolase activity
signaling
regulation of nitrogen compound metabolic process
recognition of pollen
Rich_factor
qvalue
2.59e-3
4.25e-2
8.24e-2
Count
20
40
Diff
down
up
up&down
D
Root (8 PM)
	WT-1	WT-2	WT-3	OE-TPP1-1	OE-TPP1-2	OE-TPP1-3
Solyc03g083960.3 (TPP1)	-0.86	-0.95	-0.90	0.66	1.00	1.06
Solyc09g092520.3 (XTH16B)	-1.22	-0.73	-0.70	1.15	0.60	0.90
Solyc07g005330.3 (GLC1)	-0.87	-0.82	-1.03	0.77	1.00	0.95
Solyc02g086700.4 (PR2L)	-0.95	-0.89	-0.86	0.62	1.05	1.03
Solyc07g055990.3 (XTH16A)	-0.94	-0.81	-0.97	1.05	0.88	0.80
Solyc03g093080.3 (XTH23)	-0.92	-0.79	-0.94	1.30	0.53	0.81
Solyc11g068440.2 (GLUB14)	-1.02	-0.99	-0.61	1.04	0.42	1.16
Solyc02g080160.4 (XTH8)	-0.91	-0.80	-1.03	0.95	0.90	0.89
Solyc12g011023.1 (XTH9)	-1.07	-0.89	-0.60	0.98	0.30	1.29
Solyc01g008620.4 (PR2)	-1.02	-0.72	-0.96	0.70	0.90	1.10
Solyc07g042550.3 (SS)	-0.88	-0.99	-0.83	1.18	0.75	0.77
Solyc12g017240.2 (XTH15A)	-1.00	-0.85	-0.85	1.18	0.74	0.78
Solyc07g056000.2 (XTH15B)	-0.95	-0.84	-0.93	1.05	0.72	0.95
Solyc03g093130.3 (XTH3)	-0.96	-0.89	-0.87	1.05	0.70	0.97
Solyc12g011030.3 (XTH16C)	-1.05	-0.74	-0.79	1.02	0.29	1.27
1.50
1.00
0.50
0.00
-0.50
-1.00
-1.50
E
2 days after germination
1 cm
CR-TPP1-5
CR-TPP1-10
WT
OE-TPP1-1
OE-TPP1-5
F
2 days after germination
Root length (cm)
WT
OE-SlTPP1-1
OE-SlTPP1-5
CR-SlTPP1-5
CR-SlTPP1-10
G
14 days after germination
2 cm
CR-SlTPP1-5
CR-SlTPP1-10
WT
OE-SlTPP1-2
OE-SlTPP1-5
H
14 days after germination
Root dry weight (mg)
I
Leaf (8 PM)
T6P content (ng/g DW)
WT
OE-TPP1-1
OE-TPP1-5
J
Root (8 PM)
K
Marker genes up-regulated by SnRK1
Solyc03g019690.1 (SPI5L)---Root
Solyc01g099840.3 (DAP3a)---Root
Solyc10g017960.2 (PP2A13)---Root
Solyc07g007930.3 (RFS6)---Root
Relative expression
Control
OE-TPP1

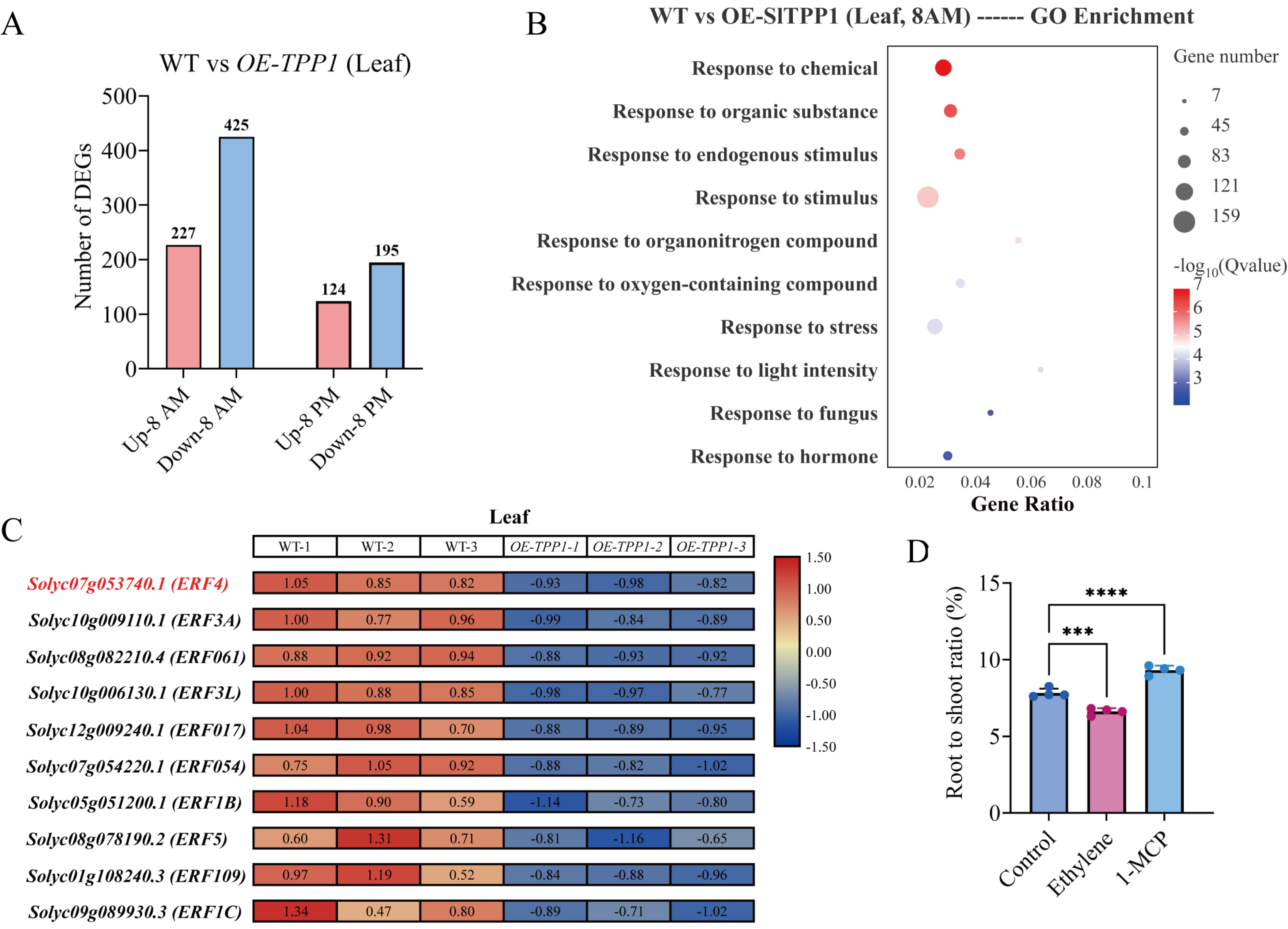
A
WT vs OE-TPP1 (Leaf)
Number of DEGs
500
400
300
200
100
0
227
425
124
195
Up-8 AM
Down-8 AM
Up-8 PM
Down-8 PM
B
WT vs OE-SlTPP1 (Leaf, 8AM) ------ GO Enrichment
Response to chemical
Response to organic substance
Response to endogenous stimulus
Response to stimulus
Response to organonitrogen compound
Response to oxygen-containing compound
Response to stress
Response to light intensity
Response to fungus
Response to hormone
0.02
0.04
0.06
0.08
0.1
Gene Ratio
Gene number
7
45
83
121
159
-log10(Qvalue)
7
6
5
4
3
C
Leaf
WT-1
WT-2
WT-3
OE-TPP1-1
OE-TPP1-2
OE-TPP1-3
Solyc07g053740.1 (ERF4) 1.05 0.85 0.82 -0.93 -0.98 -0.82
Solyc10g009110.1 (ERF3A) 1.00 0.77 0.96 -0.99 -0.84 -0.89
Solyc08g082210.4 (ERF061) 0.88 0.92 0.94 -0.88 -0.93 -0.92
Solyc10g006130.1 (ERF3L) 1.00 0.88 0.85 -0.98 -0.97 -0.77
Solyc12g009240.1 (ERF017) 1.04 0.98 0.70 -0.88 -0.89 -0.95
Solyc07g054220.1 (ERF054) 0.75 1.05 0.92 -0.88 -0.82 -1.02
Solyc05g051200.1 (ERF1B) 1.18 0.90 0.59 -1.14 -0.73 -0.80
Solyc08g078190.2 (ERF5) 0.60 1.31 0.71 -0.81 -1.16 -0.65
Solyc01g108240.3 (ERF109) 0.97 1.19 0.52 -0.84 -0.88 -0.96
Solyc09g089930.3 (ERF1C) 1.34 0.47 0.80 -0.89 -0.71 -1.02
1.50
1.00
0.50
0.00
-0.50
-1.00
-1.50
D
Root to shoot ratio (%)
15
10
5
0
****
***
Control
Ethylene
1-MCP

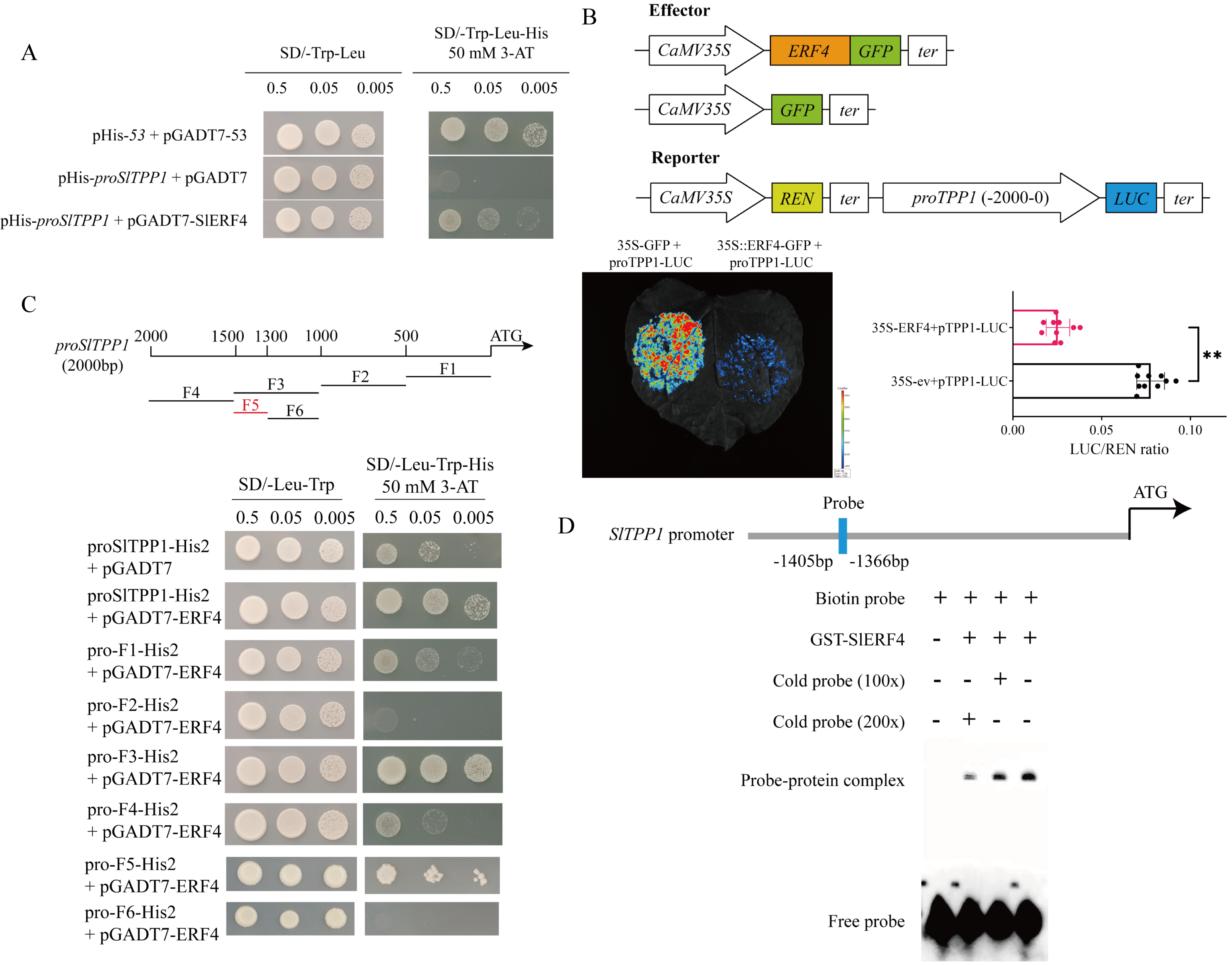
A
SD/-Trp-Leu
SD/-Trp-Leu-His
50 mM 3-AT
0.5 0.05 0.005
0.5 0.05 0.005
pHis-53 + pGADT7-53
pHis-proSlTPP1 + pGADT7
pHis-proSlTPP1 + pGADT7-SlERF4
B
Effector
CaMV35S
ERF4
GFP
ter
CaMV35S
GFP
ter
Reporter
CaMV35S
REN
ter
proTPP1 (-2000-0)
LUC
ter
35S-GFP +
proTPP1-LUC
35S::ERF4-GFP +
proTPP1-LUC
35S-ERF4+pTPP1-LUC
35S-ev+pTPP1-LUC
**
0.00
0.05
0.10
LUC/REN ratio
C
proSlTPP1
(2000bp)
2000
1500
1300
1000
500
ATG
F1
F2
F3
F4
F5
F6
SD/-Leu-Trp
SD/-Leu-Trp-His
50 mM 3-AT
0.5 0.05 0.005
0.5 0.05 0.005
proSlTPP1-His2
+ pGADT7
proSlTPP1-His2
+ pGADT7-ERF4
pro-F1-His2
+ pGADT7-ERF4
pro-F2-His2
+ pGADT7-ERF4
pro-F3-His2
+ pGADT7-ERF4
pro-F4-His2
+ pGADT7-ERF4
pro-F5-His2
+ pGADT7-ERF4
pro-F6-His2
+ pGADT7-ERF4
D
Probe
ATG
SlTPP1 promoter
-1405bp
-1366bp
Biotin probe + + + +
GST-SlERF4 - + + +
Cold probe (100x) - - + -
Cold probe (200x) - + - -
Probe-protein complex
Free probe

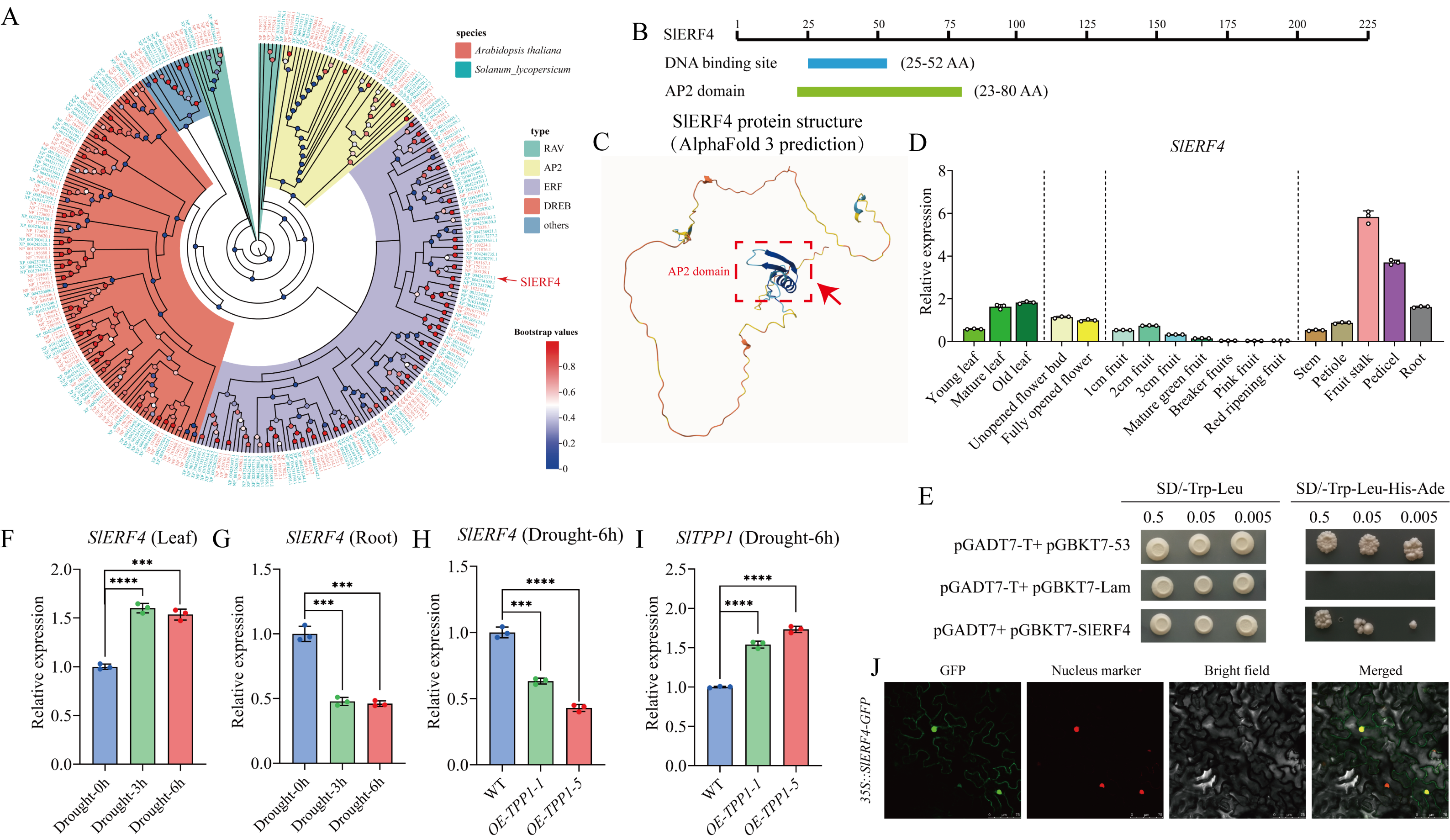
A
species
Arabidopsis thaliana
Solanum_lycopersicum
type
RAV
AP2
ERF
DREB
others
SlERF4
Bootstrap values
0.8
0.6
0.4
0.2
0
B
SlERF4
1 25 50 75 100 125 150 175 200 225
DNA binding site
(25-52 AA)
AP2 domain
(23-80 AA)
C
SlERF4 protein structure
（AlphaFold 3 prediction）
AP2 domain
D
SlERF4
Relative expression
Young leaf
Mature leaf
Old leaf
Unopened flower bud
Fully opened flower
1cm fruit
2cm fruit
3cm fruit
Mature green fruit
Breaker fruits
Pink fruit
Red ripening fruit
Stem
Petiole
Fruit stalk
Pedicel
Root
E
SD/-Trp-Leu
SD/-Trp-Leu-His-Ade
0.5 0.05 0.005
pGADT7-T+ pGBKT7-53
pGADT7-T+ pGBKT7-Lam
pGADT7+ pGBKT7-SlERF4
F
SlERF4 (Leaf)
Relative expression
Drought-0h
Drought-3h
Drought-6h
G
SlERF4 (Root)
H
SlERF4 (Drought-6h)
WT
OE-TPP1-1
OE-TPP1-5
I
SlTPP1 (Drought-6h)
J
GFP
Nucleus marker
Bright field
Merged
35S::SlERF4-GFP

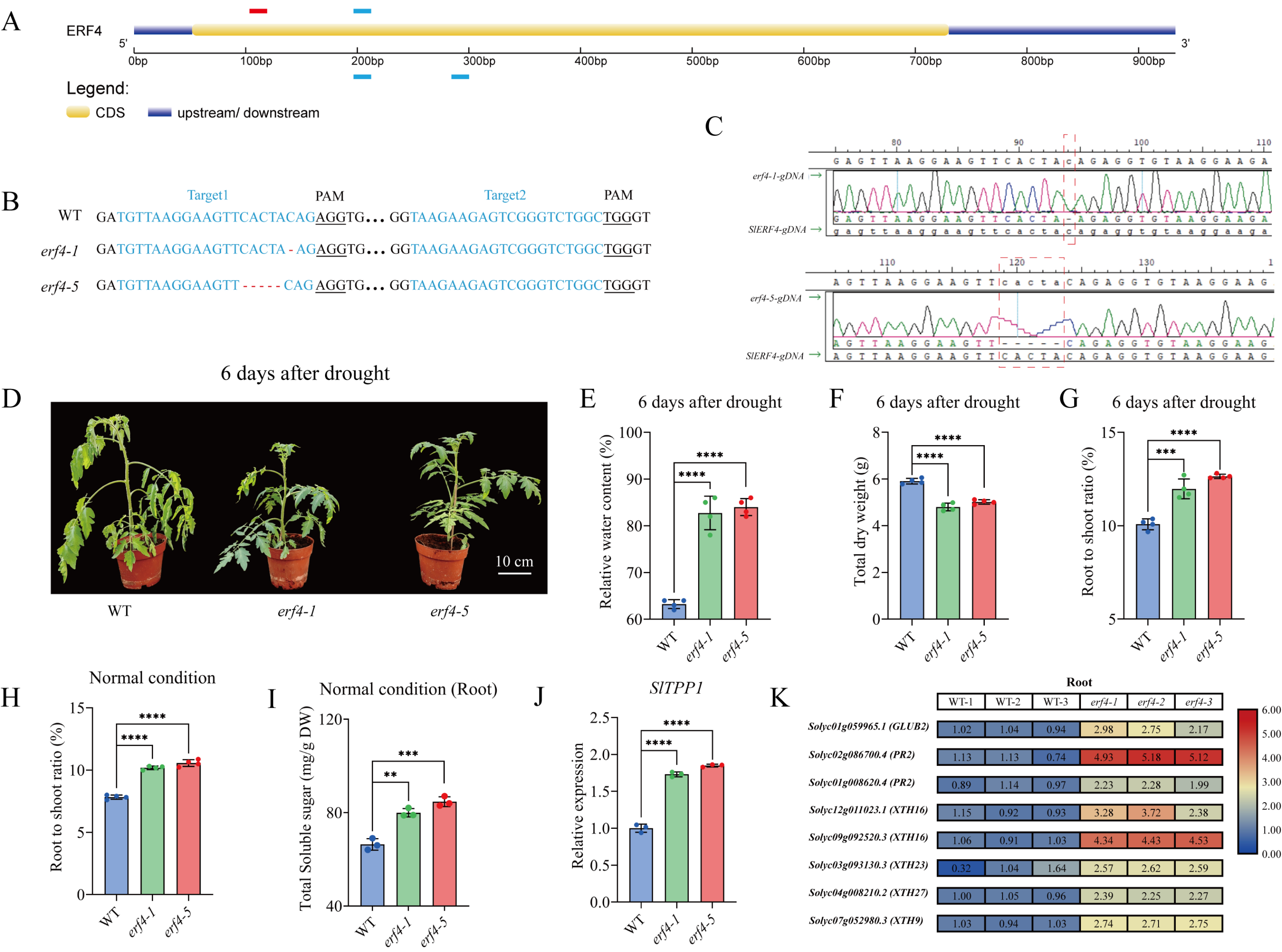

A
ERF4
5'
3'
0bp
100bp
200bp
300bp
400bp
500bp
600bp
700bp
800bp
900bp
Legend:
CDS
upstream/ downstream
B
Target1
PAM
Target2
PAM
WT GATGTTAAGGAAGTTCACTACAGAGGTG... GGTAAGAAGAGTCGGGTCTGGCTGGGT
erf4-1 GATGTTAAGGAAGTTCACTA-AGAGGTG... GGTAAGAAGAGTCGGGTCTGGCTGGGT
erf4-5 GATGTTAAGGAAGTT-----CAGAGGTG... GGTAAGAAGAGTCGGGTCTGGCTGGGT
C
erf4-1-gDNA
SlERF4-gDNA
erf4-5-gDNA
SlERF4-gDNA
D
6 days after drought
10 cm
WT
erf4-1
erf4-5
E
6 days after drought
Relative water content (%)
F
6 days after drought
Total dry weight (g)
G
6 days after drought
Root to shoot ratio (%)
H
Normal condition
Root to shoot ratio (%)
I
Normal condition (Root)
Total Soluble sugar (mg/g DW)
J
SlTPP1
Relative expression
K
Root
WT-1 WT-2 WT-3 erf4-1 erf4-2 erf4-3
Solyc01g059965.1 (GLUB2) 1.02 1.04 0.94 2.98 2.75 2.17
Solyc02g086700.4 (PR2) 1.13 1.13 0.74 4.93 5.18 5.12
Solyc01g008620.4 (PR2) 0.89 1.14 0.97 2.23 2.28 1.99
Solyc12g011023.1 (XTH16) 1.15 0.92 0.93 3.28 3.72 2.38
Solyc09g092520.3 (XTH16) 1.06 0.91 1.03 4.34 4.43 4.53
Solyc03g093130.3 (XTH23) 0.32 1.04 1.64 2.57 2.62 2.59
Solyc04g008210.2 (XTH27) 1.00 1.05 0.96 2.39 2.25 2.27
Solyc07g052980.3 (XTH9) 1.03 0.94 1.03 2.74 2.71 2.75

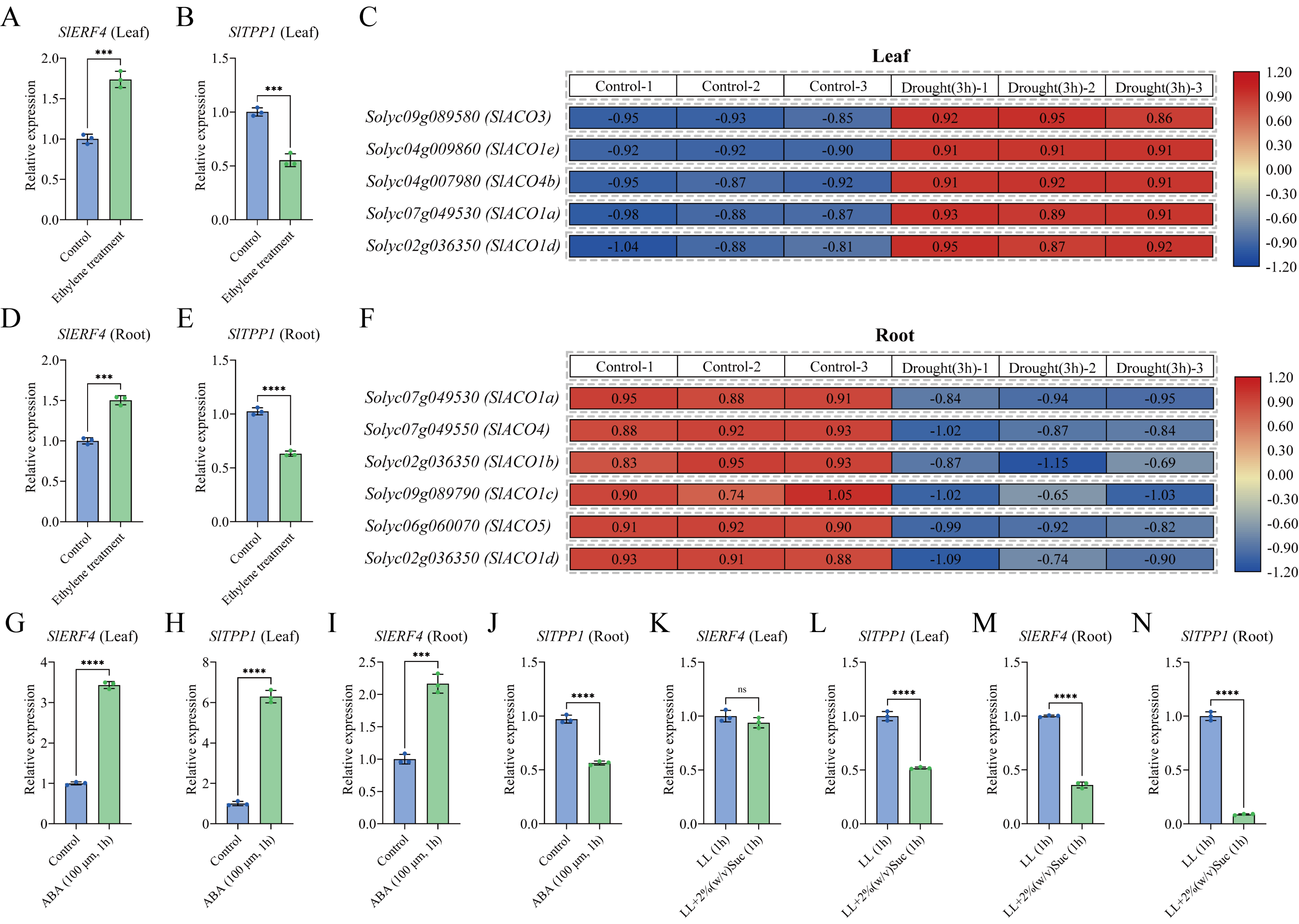
A
SlERF4 (Leaf)
Relative expression
Control
Ethylene treatment
***
B
SlTPP1 (Leaf)
***
C
Leaf
Control-1
Control-2
Control-3
Drought(3h)-1
Drought(3h)-2
Drought(3h)-3
Solyc09g089580 (SlACO3) -0.95 -0.93 -0.85 0.92 0.95 0.86
Solyc04g009860 (SlACO1e) -0.92 -0.92 -0.90 0.91 0.91 0.91
Solyc04g007980 (SlACO4b) -0.95 -0.87 -0.92 0.91 0.92 0.91
Solyc07g049530 (SlACO1a) -0.98 -0.88 -0.87 0.93 0.89 0.91
Solyc02g036350 (SlACO1d) -1.04 -0.88 -0.81 0.95 0.87 0.92
1.20
0.90
0.60
0.30
0.00
-0.30
-0.60
-0.90
-1.20
D
SlERF4 (Root)
***
E
SlTPP1 (Root)
****
F
Root
Solyc07g049530 (SlACO1a) 0.95 0.88 0.91 -0.84 -0.94 -0.95
Solyc07g049550 (SlACO4) 0.88 0.92 0.93 -1.02 -0.87 -0.84
Solyc02g036350 (SlACO1b) 0.83 0.95 0.93 -0.87 -1.15 -0.69
Solyc09g089790 (SlACO1c) 0.90 0.74 1.05 -1.02 -0.65 -1.03
Solyc06g060070 (SlACO5) 0.91 0.92 0.90 -0.99 -0.92 -0.82
Solyc02g036350 (SlACO1d) 0.93 0.91 0.88 -1.09 -0.74 -0.90
G
SlERF4 (Leaf)
****
ABA (100 μm, 1h)
H
SlTPP1 (Leaf)
****
I
SlERF4 (Root)
***
J
SlTPP1 (Root)
****
K
SlERF4 (Leaf)
ns
LL (1h)
LL+2%(w/v)Suc (1h)
L
SlTPP1 (Leaf)
****
M
SlERF4 (Root)
****
N
SlTPP1 (Root)
****

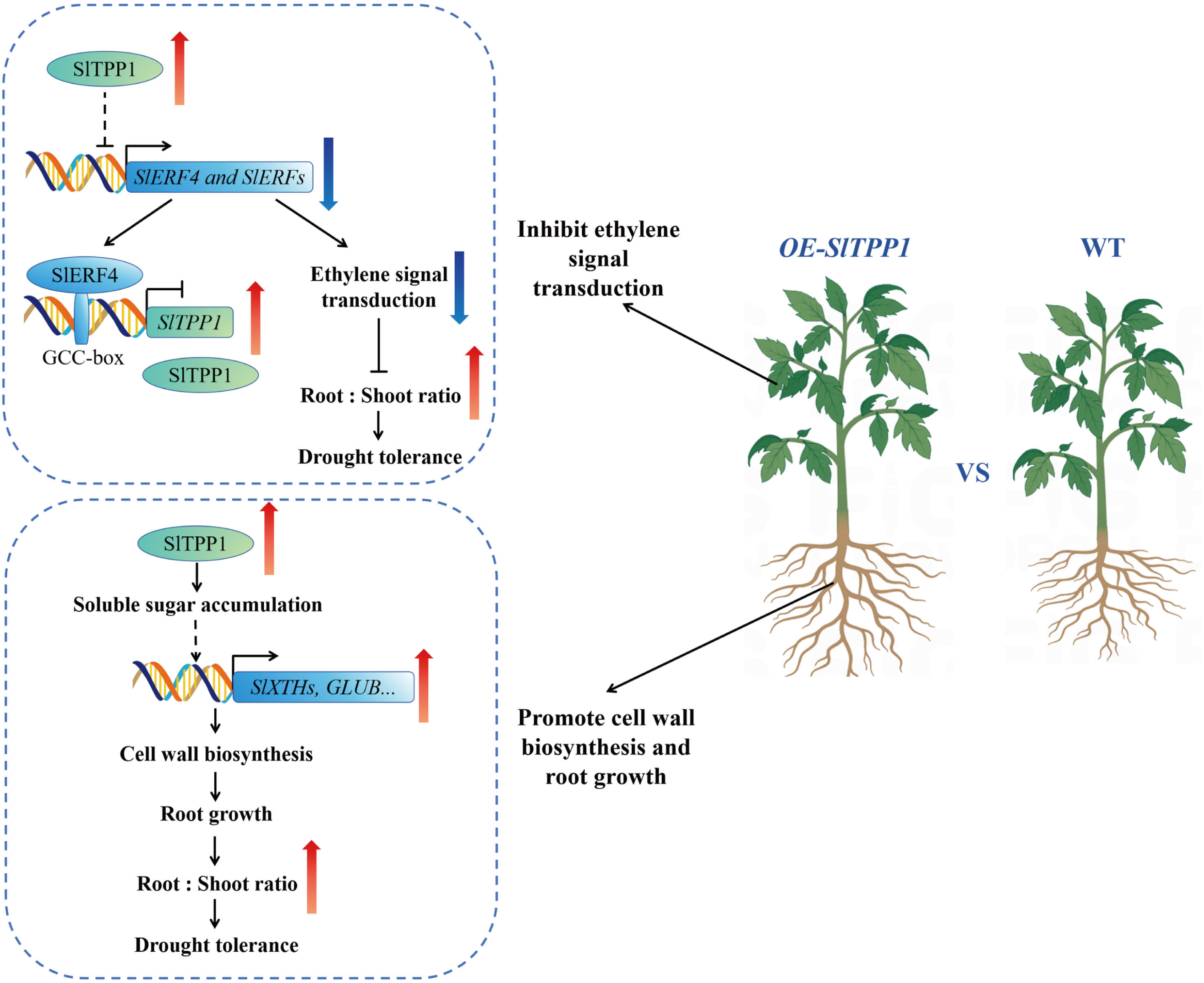
SlTPP1
SlERF4 and SlERFs
SlERF4
SlTPP1
GCC-box
SlTPP1
Ethylene signal transduction
Root : Shoot ratio
Drought tolerance
SlTPP1
Soluble sugar accumulation
SlXTHs, GLUB...
Cell wall biosynthesis
Root growth
Root : Shoot ratio
Drought tolerance
Inhibit ethylene signal transduction
OE-SlTPP1
WT
VS
Promote cell wall biosynthesis and root growth
