# Supplementary material for: A SlERF4–SlTPP1 module enhances drought tolerance in tomato by increasing the root/shoot ratio
Source: Hortic Res. 2026 Mar 2;13(6):uhag070. doi: 10.1093/hr/uhag070 (PMC13253350; doi:10.1093/hr/uhag070)
Supplement: Web_Material_uhag070 [file web_material_uhag070.zip › Supplementary data - clean-version.docx]

**Article title**: A SlERF4-SlTPP1 module enhances drought tolerance in tomato by increasing root:shoot ratio

The following Supporting Information is available for this article:

**Fig. S1** Expression profiles of *SlTPP* genes in tomato leaves under drought stress at 0 h, 3 h, and 6 h.

**Fig. S2** qRT-PCR validation of the expression levels of differentially expressed ERF transcription factor genes in leaves of WT and *OE-TPP1* transgenic tomato plants.

**Table S1** List of primers used in this study.

**Table S2** List of gene accession used in this study.


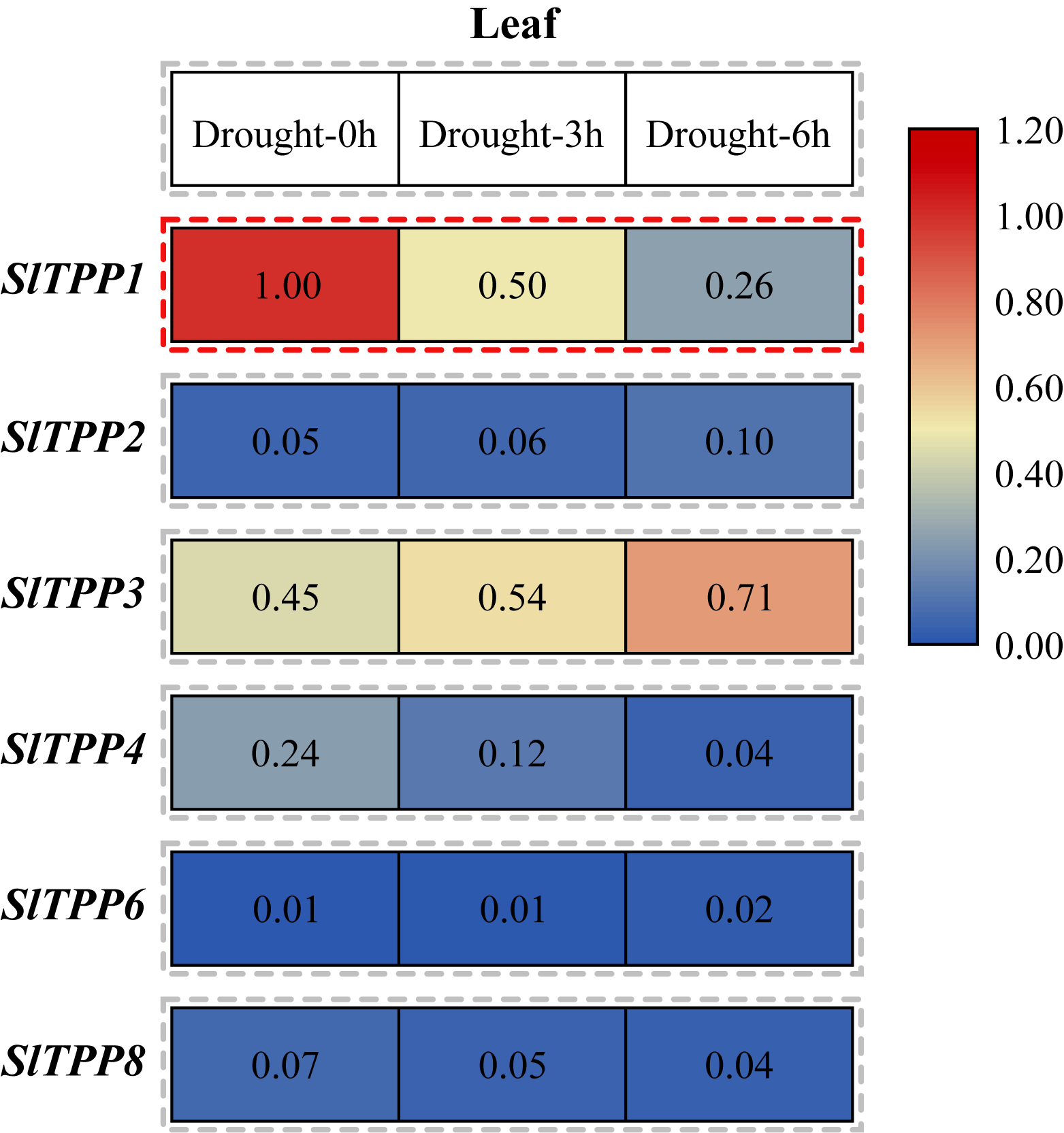


**Fig. S1** Expression profiles of *SlTPP* genes in tomato leaves under drought stress at 0 h, 3 h, and 6 h. The values in the boxes indicate the relative expression level of each gene.


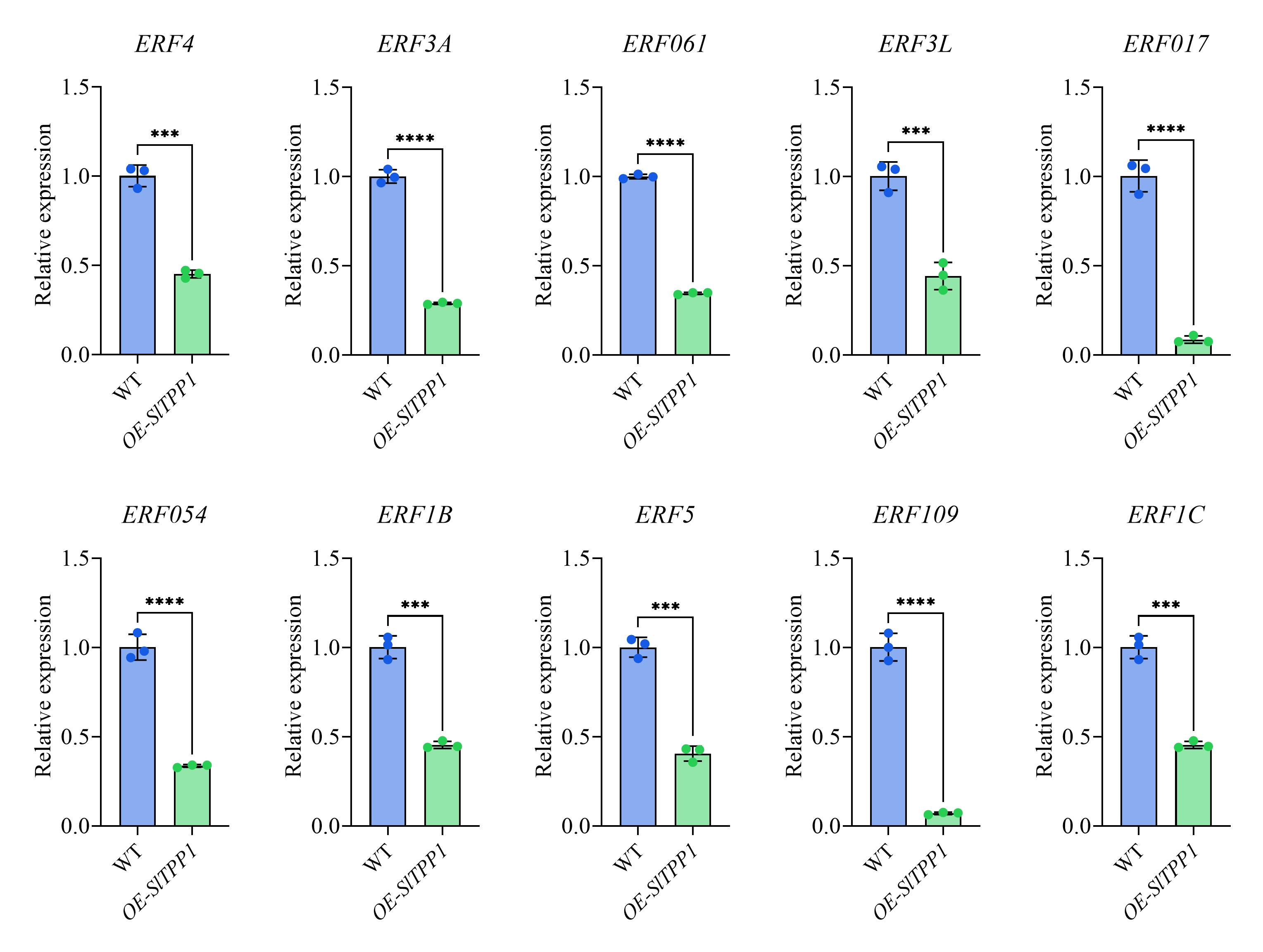


**Fig. S2** qRT-PCR validation of the expression levels of differentially expressed ERF transcription factor genes in leaves of WT and *OE-TPP1* transgenic tomato plants. Data are shown as means ± SD (n = 3). Statistical significance was analyzed using two-tailed Student’s t-test (*P < 0.05, **P < 0.01, ***P < 0.001, ****P < 0.0001).

**Table S1** List of primers used in this study.

| Name | Sequence（5'-3'） | Application |
| --- | --- | --- |
| CR-ERF4-F1 | ATATATGGTCTCGTTTGAAGAAGAGTCGGGTCTGGCGTTTTAGAGCTAGAAATAGC | CRISPR/Cas9 vector construction |
| CR-ERF4-R1 | ATTATTGGTCTCGTTAACAAACTACACTGTTAGATTC |  |
| CR-ERF4-F2 | ATATATGGTCTCGTTAAGGAAGTTCACTACAGGTTTTAGAGCTAGAAATAGC |  |
| CR-ERF4-R2 | ATTATTGGTCTCGGTCTCAAACTACACTGTTAGATTC |  |
| CR-ERF4-F3 | ATATATGGTCTCGAGACCCGACTCTTCTTACCGTTTTAGAGCTAGAAATAGC |  |
| CR-ERF4-R3 | ATTATTGGTCTCGAAACTTCCGGCGGAGATGAATAACAAACTACACTGTTAGATTC |  |
| pHis-proSlTPP1-F | gactcactatagggcgaattcTATATATATATTTTTTTTTTTCAACATTGGTAA | YIH assay |
| pHis-proSlTPP1-R | gattcgcgaacgcgtgagctcTGTGCCTAGTATCTTTTTTATGGCA |  |
| pAGDT7-SlERF4-F | gccatggaggccagtgaattcATGGCTGTGAAAGATAAGGCTGT |  |
| pAGDT7-SlERF4-R | cagctcgagctcgatggatccTTAAACTTCCATAGGTGGCGCA |  |
| pHis-proSlTPP1(-500 - -1)-F1-F | gactcactatagggcgaattcGATCTTTCCCATTTTCCTGGTTG |  |
| pHis-proSlTPP1(-500 - -1)-F1-R | gattcgcgaacgcgtgagctcTGTGCCTAGTATCTTTTTTATGGCA |  |
| pHis-proSlTPP1(-1000 - -500)-F2-F | gactcactatagggcgaattcCATGATTCTTGGTTTATCTTCAATGTC |  |
| pHis-proSlTPP1(-1000 - -500)-F2-R | gattcgcgaacgcgtgagctcGCACCCAAAAAAAAGAAAAGCA |  |
| pHis-proSlTPP1(-1500 - -1000)-F3-F | gactcactatagggcgaattcTATTTTTCGGGACTCTGTTTTTTG |  |
| pHis-proSlTPP1(-1500 - -1000)-F3-R | gattcgcgaacgcgtgagctcCTAAATACACATTTCTCCATATTATAGTTGC |  |
| pHis-proSlTPP1(-2000 - -1500)-F4-F | gactcactatagggcgaattcTATATATATATTTTTTTTTTTCAACATTGGTAA |  |
| pHis-proSlTPP1(-2000 - -1500)-F4-R | gattcgcgaacgcgtgagctcAAAATTTCTTTATCAAAACGTAATATGTACTAA |  |
| pHis-proSlTPP1(-1500 - -1300)-F5-F | gactcactatagggcgaattcTATTTTTCGGGACTCTGTTTTTTG |  |
| pHis-proSlTPP1(-1500 - -1300)-F5-R | gattcgcgaacgcgtgagctcACATATATATCACATCATACACAGCTCCA |  |
| pHis-proSlTPP1(-1300 - -1000)-F6-F | gactcactatagggcgaattcCATTTTTGGATATCATGTTCATCTGTC |  |
| pHis-proSlTPP1(-1300 - -1000)-F6-R | gattcgcgaacgcgtgagctcCTAAATACACATTTCTCCATATTATAGTTGC |  |
| 35S::ERF4-GFP-F | acgggggactcttgaggatccATGGCTGTGAAAGATAAGGCTGT | Subcellular localization |
| 35S::ERF4-GFP-R | ccgggtaccgagctcgaattcAACTTCCATAGGTGGCGCAA |  |
| proTPP1-LUC-F | gggccccccctcgaggtcgacTATATATATATTTTTTTTTTTCAACATTGGTAA | Dual-Luciferase Reporter Assay |
| proTPP1-LUC-R | cgctctagaactagtggatccTGTGCCTAGTATCTTTTTTATGGCA |  |
| GST-ERF4-F | ccgcgtggatccccggaattcATGGCTGTGAAAGATAAGGCTGT | EMSA assay |
| GST-ERF4-R | gatgcggccgctcgagtcgacTTAAACTTCCATAGGTGGCGCA |  |
| pGBKT7-ERF4-F | atggccatggaggccgaattcATGGCTGTGAAAGATAAGGCTGT | Y2H assay |
| pGBKT7-ERF4-R | ccgctgcaggtcgacggatccAACTTCCATAGGTGGCGCAA |  |
| CR-ERF4-JC-F | CACCAATTCAAAAAATCGTATCAGC | Genotype identification of mutant plants |
| CR-ERF4-JC-R | TCCCATAATGTTTCTCTTCCACAA |  |
| Actin-qF | ATCCCAAGGCCAACAGAGAG | qRT-PCR |
| Actin-qR | CGACCGCTAGCATACAGAGA |  |
| TPP1-qF | TGGCCGGAAGGTTTTAGAAGT |  |
| TPP1-qR | ATAAGCACCTCCGCTCCCTA |  |
| TPP2-qF | TAGTGGGAGGTGCAGAGACA |  |
| TPP2-qR | GCAACGGAAATGCACAGACA |  |
| TPP3-qF | TGCTCGAATCACTTGGATATGCT |  |
| TPP3-qR | TTCCACTCAACCAAACGTCG |  |
| TPP4-qF | TCCGTACATTTCCGTCGTGT |  |
| TPP4-qR | GCCTTGTCCTCTATCACGCA |  |
| TPP5-qF | TCTGTTGGGGGAGAGAACCA |  |
| TPP5-qR | GCATGACCTTTGTTCCACACA |  |
| TPP6-qF | GAAACAGCCATGACGTGCTC |  |
| TPP6-qR | CCCTCACCCATCCTGCATTT |  |
| TPP7-qF | TCTTTCTGATTCAAAGGGGCA |  |
| TPP7-qR | GTGGATGTTTGGGAGTAGCGA |  |
| TPP8-qF | AGGGGAAATGGGTTGTCGTG |  |
| TPP8-qR | TCCCCGCGTAAAACACATCA |  |
| PR2-qF | AGGACACCCTTCCGCTACT |  |
| PR2-qR | TTGGTGTTCCTGCCCCTC |  |
| XTH8-qF | GCAGGGCCATTGAGAGATGA |  |
| XTH8-qR | TCACGTCCACCACTCCCA |  |
| PR2L-qF | GGACACCCTGCTGCAACT |  |
| PR2L-qR | AGGCTTCTTTGGAGTGCCA |  |
| XTH23-qF | GGCATTGCAATTGGGGCC |  |
| XTH23-qR | AGAGAGTAAGGAGGTCGCCA |  |
| XTH3-qF | GGCATTGCAATTGGGGCC |  |
| XTH3-qR | AGAGAGTAAGGAGGTCGCCA |  |
| GLC1-qF | CTGCTGAGACAGGGTGGC |  |
| GLC1-qR | CGATCTTCCTGTGCGGCA |  |
| SS-qF | AACCGCGTGAGGAATGGG |  |
| SS-qR | AGGCAAACCGCAGCTCAT |  |
| XTH16A-qF | GCGCTTCCTCATGTGCCT |  |
| XTH16A-qR | CCCATCGGACCCTGTTCC |  |
| XTH15B-qF | GATGGAGGGCGCCTTCTC |  |
| XTH15B-qR | TGGTGACAGTGCCAGCAG |  |
| XTH16B-qF | TTGGGCCACAAGAGGAGG |  |
| XTH16B-qR | GGCTAAAGCTTTGGGCCA |  |
| GLUB14-qF | GGCAAAAGGGGCAATGGC |  |
| GLUB14-qR | CTCGTGAGGGTGTTGGCA |  |
| XTH9-qF | GCAATGTCTCTGGCCAGC |  |
| XTH9-qR | ACGCGGCAGTTGGATCAA |  |
| XTH16C-qF | GCAATGTCTCTGGCCAGC |  |
| XTH16C-qR | ACGCGGCAGTTGGATCAA |  |
| XTH15A-qF | TCAGGCCAACCGTACACG |  |
| XTH15A-qR | GTGGAACGACGAGGTGGG |  |
| ERF4-qF | GGAGAAACGGTTGTTCACGC |  |
| ERF4-qR | CCATAGGTGGCGCAAGGTTA |  |
| ERF1B-qF | AGTTAGAAAGAGGCCATGGGG |  |
| ERF1B-qR | AGTAGAGACCAAGGACCCCTC |  |
| ERF3L-qF | GCTGAAGATGCTGCCAAAGC |  |
| ERF3L-qR | GGTCTCGGTCCACTGAATGA |  |
| ERF5-qF | AGCCGTCGATTTCAAGCTCT |  |
| ERF5-qR | CTGCCGCACTCCTCTGTAAT |  |
| ERF061-qF | GCGATCCGAGTAAGTTGGGA |  |
| ERF061-qR | GGATCCTTCAAGCAATCACCA |  |
| ERF1C-qF | AGTCACATCGCATCGCGTTA |  |
| ERF1C-qR | GCGAATCCCTAACGGTCTCC |  |
| ERF054-qF | CTTATGACCGCGAGGCGTAT |  |
| ERF054-qR | TCGATCCTATCCCCGAGTCC |  |
| ERF3A-qF | GTGGAGCGAAAGCGAAAACT |  |
| ERF3A-qR | GATTCACCGCCTGTACCGAA |  |
| ERF025-qF | AGCCTCGAAAGACCACACG |  |
| ERF025-qR | GACAGAGAAGCAGGGACAGG |  |
| ERF017-qF | TTCACAGTCGTCATCGTCGT |  |
| ERF017-qR | AGTCATCGTCCTTCCGTTCC |  |
| SPI5L-qF | CGGGTGGTGACGTGTACC |  |
| SPI5L-qR | GCGTACCGATGGGTCGTC |  |
| DAP3A-qF | CCGGCCGGTACTACTCCT |  |
| DAP3A-qR | GGGCTTCTGGGTCCAACC |  |
| PP2A13-qF | GGCCTGCGTTGCTCTAGT |  |
| PP2A13-qR | CGCCGAAGAAGCTGCATG |  |
| RFS6-qF | GCACCTGGCAAGCACAAC |  |
| RFS6-qR | GGTCGACCAGGCAAACGA |  |
| TPP1-probe1-F | AGATATTATGTTGTGAAATCGGTGGTGCTGTTTCAAGTGA | EMSA assay |
| TPP1-probe1-R | TCACTTGAAACAGCACCACCGATTTCACAACATAATATCT |  |

**Table S2** List of gene accession used in this study.

| Gene ID | Symbol |
| --- | --- |
| Solyc03g078400 | Actin |
| Solyc03g083960 | TPP1 |
| Solyc05g051880 | TPP2 |
| Solyc04g054930 | TPP3 |
| Solyc04g072920 | TPP4 |
| Solyc04g082550 | TPP5 |
| Solyc06g060600 | TPP6 |
| Solyc08g079060 | TPP7 |
| Solyc03g007290 | TPP8 |
| Solyc09g092520.3 | XTH16B |
| Solyc07g005330.3 | GLC1 |
| Solyc02g086700.4 | PR2L |
| Solyc07g055990.3 | XTH16A |
| Solyc03g093080.3 | XTH23 |
| Solyc11g068440.2 | GLUB14 |
| Solyc02g080160.4 | XTH8 |
| Solyc12g011023.1 | XTH9 |
| Solyc01g008620.4 | PR2 |
| Solyc07g042550.3 | SS |
| Solyc12g017240.2 | XTH15A |
| Solyc07g056000.2 | XTH15B |
| Solyc03g093130.3 | XTH3 |
| Solyc12g011030.3 | XTH16C |
| Solyc07g053740.1 | ERF4 |
| Solyc10g009110.1 | ERF3A |
| Solyc08g082210.4 | ERF061 |
| Solyc10g006130.1 | ERF3L |
| Solyc12g009240.1 | ERF017 |
| Solyc07g054220.1 | ERF054 |
| Solyc05g051200.1 | ERF1B |
| Solyc08g078190.2 | ERF5 |
| Solyc01g108240.3 | ERF109 |
| Solyc09g089930.3 | ERF1B |
| Solyc07g049530.3 | ACO1a |
| Solyc02g036350.3 | ACO1b |
| Solyc09g089790.3 | ACO1c |
| Solyc02g036350.3 | ACO1d |
| Solyc04g009860.4 | ACO1e |
| Solyc09g089580.4 | ACO3 |
| Solyc07g049550.3 | ACO4 |
| Solyc04g007980.3 | ACO4b |
| Solyc06g060070.3 | ACO5 |
| Solyc03g019690.1 | SPI5L |
| Solyc01g099840.3 | DAP3A |
| Solyc10g017960.2 | PP2A13 |
| Solyc07g007930.3 | RFS6 |
