# Supplementary figures and images for: A SlERF4–SlTPP1 module enhances drought tolerance in tomato by increasing the root/shoot ratio
Source: Hortic Res. 2026 Mar 2;13(6):uhag070. doi: 10.1093/hr/uhag070 (PMC13253350; doi:10.1093/hr/uhag070)

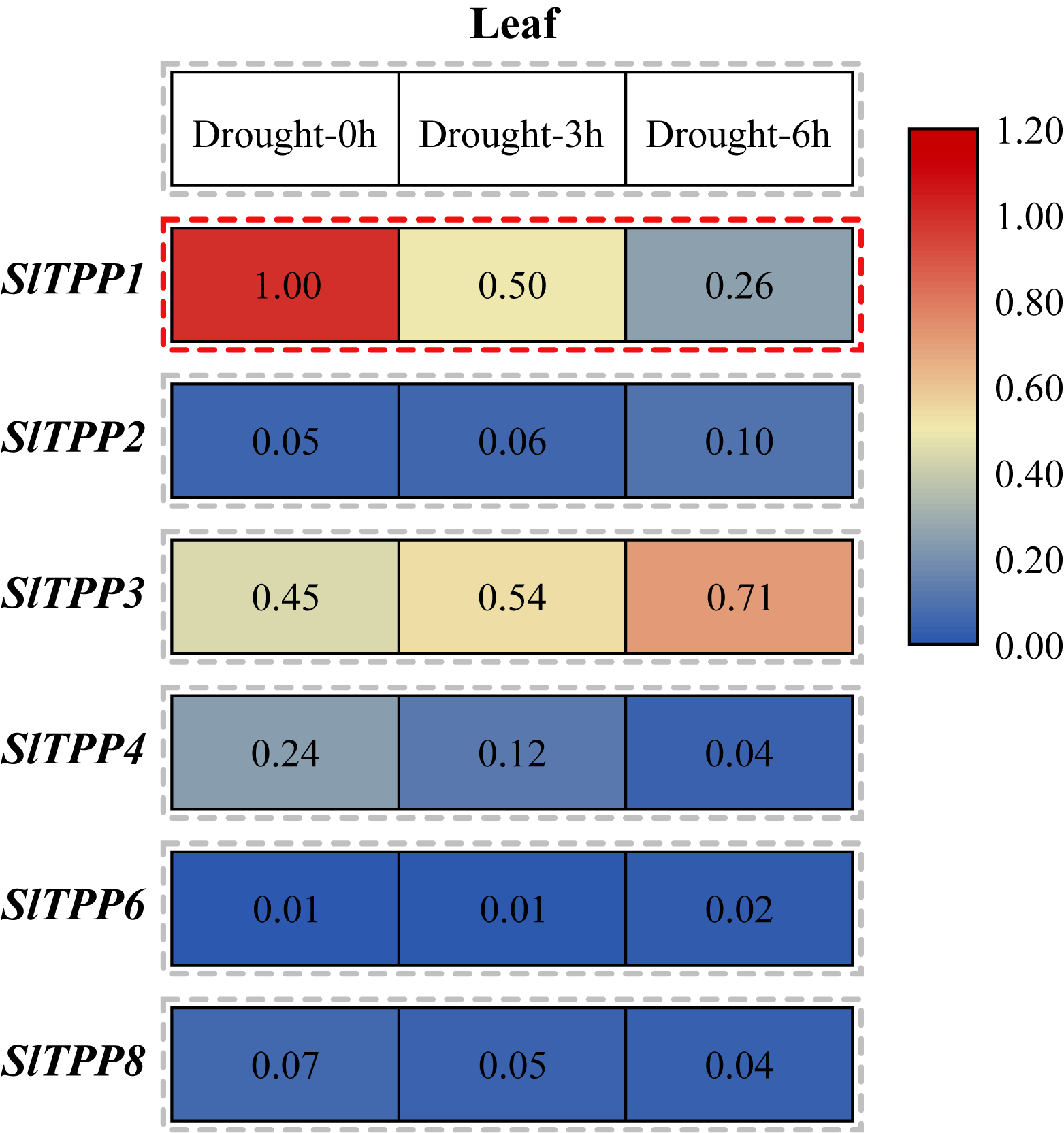

Supplement: Web_Material_uhag070 [file web_material_uhag070.zip › Fig. S1.tif]

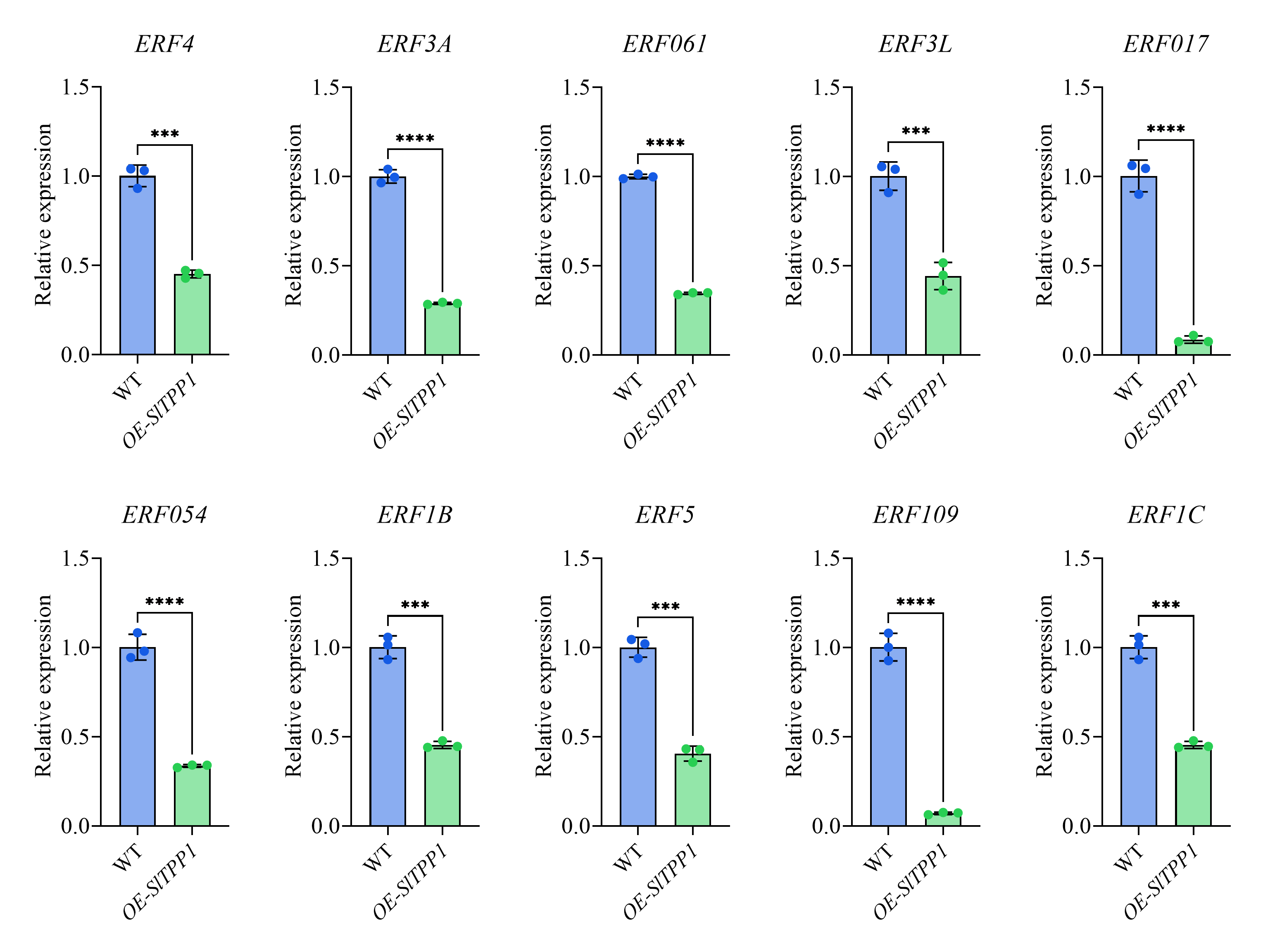

Supplement: Web_Material_uhag070 [file web_material_uhag070.zip › Fig. S2.tif]
